# A Review of Recent Advances in Natural Polymer-Based Scaffolds for Musculoskeletal Tissue Engineering

**DOI:** 10.3390/polym14102097

**Published:** 2022-05-20

**Authors:** Jingzhi Fan, Keyvan Abedi-Dorcheh, Asma Sadat Vaziri, Fereshteh Kazemi-Aghdam, Saeed Rafieyan, Masoume Sohrabinejad, Mina Ghorbani, Fatemeh Rastegar Adib, Zahra Ghasemi, Kristaps Klavins, Vahid Jahed

**Affiliations:** 1 Rudolfs Cimdins Riga Biomaterials Innovations and Development Centre of RTU, Institute of General Chemical Engineering, Faculty of Materials Science and Applied Chemistry, Riga Technical University, Pulka St 3, LV-1007 Riga, Latvia; jingzhi.fan@rtu.lv; 2 Baltic Biomaterials Centre of Excellence, Headquarters at Riga Technical University, Pulka St 3, LV-1007 Riga, Latvia; 3 Biomedical Engineering Division, Faculty of Chemical Engineering, Tarbiat Modares University, Tehran 14117-13116, Iran; keyvan.abedi@modares.ac.ir (K.A.-D.); asma.vaziri86@gmail.com (A.S.V.); fereshtehkazemiaghdam@gmail.com (F.K.-A.); saeed21041374@gmail.com (S.R.); m.sohrabinejad@modares.ac.ir (M.S.); mina.ghorbani2020@gmail.com (M.G.); rastegar.ft@gmail.com (F.R.A.); ghasemi.zh@yahoo.com (Z.G.)

**Keywords:** natural polymers, biomaterials, biodegradable scaffolds, musculoskeletal tissue, tissue engineering

## Abstract

The musculoskeletal (MS) system consists of bone, cartilage, tendon, ligament, and skeletal muscle, which forms the basic framework of the human body. This system plays a vital role in appropriate body functions, including movement, the protection of internal organs, support, hematopoiesis, and postural stability. Therefore, it is understandable that the damage or loss of MS tissues significantly reduces the quality of life and limits mobility. Tissue engineering and its applications in the healthcare industry have been rapidly growing over the past few decades. Tissue engineering has made significant contributions toward developing new therapeutic strategies for the treatment of MS defects and relevant disease. Among various biomaterials used for tissue engineering, natural polymers offer superior properties that promote optimal cell interaction and desired biological function. Natural polymers have similarity with the native ECM, including enzymatic degradation, bio-resorb and non-toxic degradation products, ability to conjugate with various agents, and high chemical versatility, biocompatibility, and bioactivity that promote optimal cell interaction and desired biological functions. This review summarizes recent advances in applying natural-based scaffolds for musculoskeletal tissue engineering.

## 1. Introduction

The musculoskeletal (MS) system consists of bone, cartilage, tendon, ligament, and skeletal muscle, which form the basic framework of the human body. The damage or loss of MS-related tissues significantly affects the quality of life. MS disorders can be caused by old age, traumatic events, autoimmune and degenerative diseases. According to the WHO report, between one in three and one in five people worldwide suffer from the mentioned disorders, which have the most persistent pain in non-cancerous cases [1,2,3,4].

The self-healing potential of MS-related tissues during injury depends on tissue type and the degree of damage and inflammation. Whereas bones and skeletal muscles have an adequate intrinsic ability for self-healing in minor injuries, the self-repair of severe injuries and injuries to other MS-related tissues needs clinical interventions for complete healing [5,6,7]. In these cases, donor grafting, a conventional clinical treatment, is limited due to prolonged recovery time, risk of infection, immunological rejection, and donor site morbidity. To address these challenges, MS tissue engineering has emerged and developed as an alternative therapeutic option to fully recover the patient by boosting the spontaneously healing potential of the native tissues [8,9,10].

Tissue engineering provides an efficient approach for repairing damaged or lost tissues by combining scaffolds, cells, and signaling molecules. To this end, a scaffold is an essential part that can accommodate stem cells and biological cues such as small molecules and growth factors. A scaffold-based strategy can be applied as a local tool to accelerate the regeneration process [11,12]. Ideally, tissue-engineered scaffolds must be biocompatible and non-immunogenic, and their degradation rate is commensurate with the re-formation of new tissue. Additionally, these scaffolds should have the appropriate surface chemistry for cell adhesion and the desired porosity for the transport of oxygen, nutrients, and metabolic wastes. Furthermore, their mechanical properties should correspond to the host tissue strength to sustain the regeneration of tissue during the healing process and induce targeted stem cell differentiation to the host cells [13,14].

The fabrication of an artificial microenvironment with a suitable polymer should mimic the host tissue’s native extracellular matrix (ECM) to guarantee successful tissue regeneration. ECM is a dynamic three-dimensional structure composed of glycoproteins and glycosaminoglycans, which have a tissue-specific proportion of these components and architecture. This non-cellular component acts as a physical scaffold for cells and controls cellular behavior such as homeostasis, adherent, proliferation, and cell differentiation through biochemical and biomechanical signals. Therefore, host ECM simulation becomes the most crucial part of scaffold design, especially for the scaffolds with incorporated cells [15].

**Figure 1:**
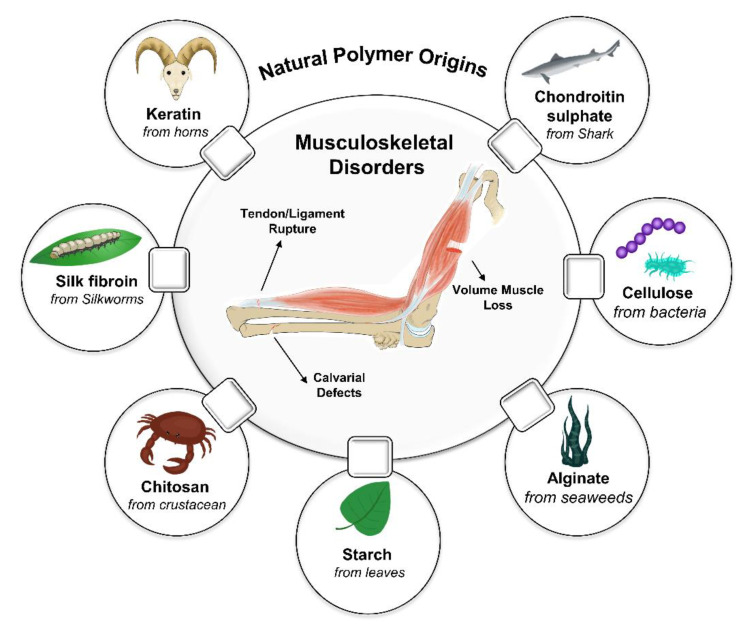
Schematic diagram of natural sources of natural polymers and their application in musculoskeletal tissue disorders.

Natural polymers are desirable among various biomaterials used for scaffolding, such as alloys, ceramics, and polymers. Figure 1 shows the natural-based polymers applied for tissue engineering and their extraction source. Recently, various types of natural-based polymer scaffolds with different architectures, including hydrogel, fibrous, solid porous, and a composite with decellularized tissue, have been developed for MS tissue engineering applications [5,16,17,18,19]. They offer superior biocompatibility, bioactivity, and high chemical versatility for desired biological function. Based on their sources, natural polymers can be classified into two main categories: (i) non-mammalian, which includes Marine algae (Carrageenans, Agarose, Alginate), crustacean (chitosan/chitin), insects (silk fibrin), plants (starch), microorganisms (xanthan gum, gellan gum, dextran), (ii) mammalian-based, including proteins (collagen, fibrin, elastin) and glycosaminoglycans (chondroitin sulfate, hyaluronan, and heparin). The basic structures of these polymers include proteins, polypeptides, and polysaccharides, which can mimic their various functions in the native ECM when registered as an engineered scaffold.

Hydrogels are hydrophilic three-dimensional networks made of physically associated or chemically crosslinked polymer chains that can uptake high amounts of water and biological fluids. A key feature of hydrogels is the structural similarities to the ECM due to their soft and flexible nature. Their physicochemical properties can be easily tailored, allowing them to be used in various tissue reconstruction applications [20,21].

Fiber scaffolds with a high length-to-width ratio are among the most attractive polymeric constructions in the tissue engineering field due to resembling the fibrous microstructure of muscles and connective tissues cartilage, bone, ligament, and tendon. This fibril architecture in various nano- and microscales plays an essential role in the mechanical properties and regulation of cell differentiation behavior [22,23].

Decellularized scaffolds prepared by removing cellular contents from native tissues or organs provide an ideal scaffold by preserving the architecture, components, and ligands of native ECM. Tissue-engineered grafts can be ex vivo re-cellularized with stem cells and applied for organ transplantation to reduce immune rejection [8,24].

Solid porous scaffolds serve as a three-dimensional matrix with interconnected pores and high porosity. This interconnected porous structure is essential for high-cell density culture and tissue growth, especially for organ angiogenesis and bone formation. Furthermore, their surface-to-volume ratio, crystallinity, porosity, and the size, shape, and interconnection of pores can be controlled to adapt to different application requirements in engineering various tissue types [25,26].

In this review, we describe the ECM structure corresponding to every distinct part of musculoskeletal tissue, which is followed by short explanations of what disorders are associated with them. We provide a concise review of recent advancements in natural-based scaffolds for each musculoskeletal tissue type and shortly discuss challenges and future directions.

It should be pointed out that the discussion about cartilage requires a separate article due to the diversity of cartilage types (including hyaline, fibrocartilage, and elastic cartilage) involved in the musculoskeletal tissue. Hence, this manuscript focuses on recent bone, tendon, ligament, and skeletal muscle tissue engineering advances. The key properties of these natural polymers are summarized in Table 1.

## 2. Bone

Bone tissue consists of different types of cells and an extracellular matrix, which is mainly composed of collagen proteins. The major functions of bone include structural support, mechanical movement, hemopoiesis, and organ protection; it also acts as a body resource of calcium and phosphate ions [82,83]. The resorption and formation of bone are tightly regulated and orchestrated under bone homeostasis to keep skeletal integrity [84]. Bone tissue contains different types of cells, including osteoblasts, osteoclasts, and osteocytes. Osteoblasts and osteocytes originate from mesenchymal stem cells (MSCs), while osteoclasts are derived from hematopoietic stem cells. Ninety percent (90%) of the bone cell population includes osteocytes, which act as the primary cells for bone formation, mineralization, and regulating cell signaling. During the physiological process of bone remodeling, the damaged bone is resorbed by osteoclasts, and new bone, which is generated by osteoblasts, is replaced [84]. There is a balance between osteoclast-mediated bone resorption and osteoblast-mediated bone formation in healthy bone, which is controlled by several coordinated signaling mechanisms. However, under certain pathological conditions, an imbalance between these two processes may occur, leading to bone diseases.

### 2.1. Bone Extracellular Matrix

Type I collagen makes up most of the ECM in bone, and its orientation directly impacts its mechanical properties. Collagen fibers arranged in a uniform and parallel pattern reinforce the bone [85]. Apatite mineral crystallites comprise 65% of the total bone mass as the inorganic part of the ECM [86]. The direction of collagen fibrils and apatite crystals in ECM creates diverse mechanical properties in different bone types, e.g., being co-aligned in a direction makes the bone stiff and tight [87,88]. Other important non-cellular components of bone ECM are glycosaminoglycans, proteoglycans, cell adhesion cytokines, and key growth factors [89].

### 2.2. Bone Structure

**Figure 2:**
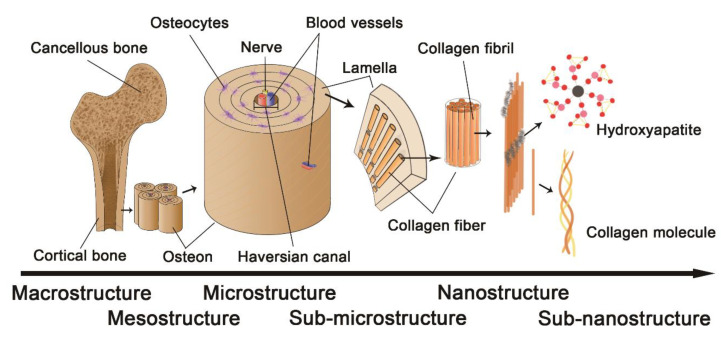
The hierarchical structure of bone.

The complex and hierarchical bone structure is divided into different parts based on macroscale (cancellous bone and cortical bone), microscale (Haversian canals, osteons), sub-microscale (single layer of lamella with collagen fibers), nanoscale (collagen fibrils), and sub-nanoscale (minerals, collagen molecule) (Figure 2). Spongy cancellous bone, which is distributed on the surface of the bone, is made up of intertwined bone trabeculae. Cortical bone (compact bone) is strong in compression and distortion due to its high density. Osteons, which are cylinders that contain osteocytes, are placed parallel to the shaft of the bone tube. Each osteon consists of lamellas surrounding the Haversian canal, containing blood vessels and fiber arrays as its subunits, containing mineralized collagen fibrils made of adjacent blocks adhered by crosslinkers. Collagen molecules comprise triple helix chains that coil each other and are stabilized by internal bonds. Crystallized apatite, the inorganic substance, is located between collagen fibrils [90].

The hierarchical structure in the cortical bone can be divided into six levels: (1) Macrostructure level (>10 mm), which consists of cortical and trabecular bone types, (2) Mesostructure level (0.5–10 mm), where osteons array together, (3) Microstructure level (10–500 µm), where a single osteon contains interstitial lamella, (4) Sub-microstructural level (1–10 µm), which is also a single lamella, (5) Nanostructure level (10–1000 nm), which is a multiphase nanocomposite consisting of an organic phase, inorganic phase, and water, and (6) Sub-nanostructure (<10 nm) in which molecules can be analyzed separately.

### 2.3. Bone Diseases

There are many bone diseases, usually leading to fractures and defects. Osteogenesis imperfecta is generated by a defect in collagen and results in less organized bone; therefore, the bone fails as it is faced with only minimal amounts of tension. Osteoporosis, the most common bone disease, is characterized by decreased bone mass and deterioration of bone structure [91]. The defects in osteoclastic bone resorption cause osteopetrosis disease, which, despite increasing bone mass, will be followed by skeletal fragility. Osteosarcoma is a common bone tumor that mainly occurs in the large bones and the knee [92].

Conventional clinical therapies for bone filling, such as autologous and allogeneic bone grafts, suffer from several shortcomings, i.e., immune rejection, infection, insufficient or missing osseointegration, and lack of a donor. Bone tissue engineering has emerged as a novel method to hinder the mentioned risks. The new approaches for regenerating damaged bone are developed using the tissue engineering triangle: signaling molecules, cells, and scaffolds. Below, we summarize recent examples of natural-based polymers that have been used for bone tissue engineering.

### 2.4. Natural-Based Scaffolds for Bone Tissue Engineering

#### 2.4.1. Collagen

As the main organic matrix of bone, type I collagen has superior bioactivity and biocompatibility as implants. However, the mechanical properties of collagen are not ideal for hard tissue engineering. As a result, many studies of collagen-based scaffolds have focused on improving strength, osteogenesis, and bioavailability.

Ceramics are usually used as enhancers to improve the strength of collagen-based material owing to their great mechanical strength. Among these, hydroxyapatite (HA), ꞵ-tricalcium phosphate (ꞵ-TCP), and bioactive glasses (BGs) are mainly employed with collagen for bone scaffolds. HA and ꞵ-TCP can also provide essential elements such as calcium and phosphorus for the bone matrix. Combining collagen–TCP composites with other materials has been evaluated as a biomimicking matrix and delivery vehicle of growth factors to improve their structural and biological properties [93]. ꞵ-TCP can provide good osteoconductivity and accelerate the degradation rate of the scaffold, which eventually can be replaced by a newly formed bone. The optimal ꞵ-TCP concentration should be 5–10 wt% to control the rapid release of Ca^2+^ [94]. HA is the original component of the bone matrix; therefore, its application for bone implants is widely studied. Although HA has superiorities with non-reactivity, osteoconductivity, and outstanding strength to composite collagen, the shortcomings such as the low degradation rate of HA still inhibit the development of HA/collagen materials. As a result, biphasic calcium phosphate (BCP) provides both the stability of HA and the biodegradability of ꞵ-TCP and has emerged as a promising future direction [95].

Bioactive glasses (BGs) are silica-based biomaterials that contain SiO_2_-CaO-P_2_O_5_ networks. The release of Na^+^, Ca^2+^, and Si^4+^ can trigger osteoblast proliferation and differentiation by stimulating osteogenesis. More importantly, due to the formation of silanol active sites, it has been used for tissue binding and mineralization [96]. BGs offer higher bioavailability and bioactivity due to their higher surface reactivity than HA and ꞵ-TCP [97,98]. Ferreira et al. took advantage of the bioglass and carbonate apatite composite mineralized collagen scaffold to promote human osteoblast differentiation [98]. The composites could stimulate osteoblast differentiation and mineralization in vitro without osteogenic dopants [99].

Synthetic polymers are also applied to enhance the mechanical properties of collagen. Polymers such as polylactic acid (PLA) [100], poly lactic-co-glycolic acid (PLGA) [101], and polycaprolactone (PCL) [102] are often used for collagen composites.

Nowadays, 3D printing is commonly used for polymer processing due to the rapid development of this technology. The osteoconductive and osteoinductive properties of a 3D-printed PLA/collagen scaffold were proved by in vitro biomineralization tests [100]. Dewey et al. utilized fluffy-PLGA to reinforce mineralized collagen scaffolds to form a bone mesh [101]. The in vitro tests showed that this composite could increase hMSC osteogenesis and locally inhibit osteoclast activity to accelerate bone regeneration.

The biomimetically inspired approach is a promising strategy for forming osteogenic and hematopoietic niches and shows considerable osteoinductivity by the expression of cells and bone marrow stromal cell markers. Proteins and ions are frequently applied as dopants to achieve biological purposes. Tadalafil is a phosphodiesterase (PDE) enzyme inhibitor that benefits angiogenesis by upregulating the expression of VEGF and CYR61 as well as increasing the effect of nitrous oxide (NO) and the level of cGMP. The Tadalafil/ꞵ-TCP/collagen scaffold was prepared and further implanted in vivo in a rabbit critical-size calvarial defect, and it led to accelerating osteogenesis following 6 weeks [103]. The substitution of magnesium ions (Mg^2+^) can induce angiogenesis through nitric acid production [104,105,106]. A recent study utilized magnesium as the primary material cooperated with collagen and HA to achieve a better degradation rate [107]. Copper ions (Cu^2+^) are also available for bone implants. Culturing of pre-osteoblast cells on a porous collagen/copper-doped bioactive glass scaffold showed enhanced osteogenesis and angiogenesis [108]. Furthermore, when implanted in a chick embryo ex vivo model, it exhibited potential for osteomyelitis treatment by limiting infection while enhancing angio- and osteogenesis effect [108]. Other essential trace elements in the body play important roles in bone metabolism’s anabolic and catabolic aspects. A collagen/HA porous scaffold incorporated with carboxyl-functionalized carbon nanotube (CNT) was developed to transplant MSCs in Sprague–Dawley rats with parietal bone defects [109]. After 12 weeks of implanting collagen/HA/CNT scaffold in a rat critical-sized calvarial defect model, favorable biocompatibility and biodegradability were observed. Furthermore, the utilization of CNT enhanced the mechanical strength and osteogenesis of the scaffold [109].

To offset the weaknesses of collagen, the reinforcement with ceramics and synthetic/natural-based polymers is a promising solution for bone tissue engineering.

#### 2.4.2. Gelatin

Gelatin is a hydrolyzed form of collagen derived from acid and alkali pre-treatments of bovine and porcine collagen [65]. Gelatin has significant biocompatibility due to Arg-Gly-Asp (RGD), which is available in its structure, promoting cell attachment, spreading, and proliferation. However, the poor mechanical properties prohibit its direct usage for bone defect treatments. Several studies focus on gelatin-based scaffolds incorporated with other materials to evaluate mechanical stability and the osteogenic differentiation of osteoblasts. Micro and nano-additives such as silica nanoparticles, polymer microparticles, and nano-HA can be employed to improve mechanical strength and are additionally used as controlled delivery systems for osteogenesis, angiogenesis, and drug agents [110,111]. The dopants for biological functions usually aim at improved osteoinductivity, anti-inflammatory, and antibacterial ability. A study of an alginate–TCP–gelatin porous scaffold loaded with dimethyloxalylglycine demonstrated an upregulation of angiogenesis markers [112]. Furthermore, in vivo tracking of stem cells seeded on the scaffold demonstrated considerable osteogenesis and angiogenesis potential. However, the sample’s mechanical properties from this study lacked adequate strength to regenerate large-sized bone defects fully [112]. Although incorporating bioceramics can result in osteoconduction and mechanical strength, the balance between porosity and strength is still a challenge for researchers. The strategies to solve this contradiction include improving compositions, microstructures, and processing methods. One such illustration is the gelatin–PCL–nanoHA composite scaffold prepared by electrospinning [113]. The effect of several processing parameters such as porosity, fiber diameter, pore size, and HA concentration was investigated. Three-dimensional (3D) printing is more precise than electrospinning when a complex porous structure is needed. A graphene/gelatin/chitosan/TCP composite was recently fabricated by Lu et al. through additive manufacturing [114]. The combination of various materials and 3D printing provides scaffolds with a complex 3D structure and antibacterial properties.

With many functional groups in gelatin, chemical modification is also an attractive approach to developing gelatin-based scaffolds. Gelatin methacryloyl (GelMA), a photocrosslinkable gelatin, is one of the most studied. A recent study loaded metformin into mesoporous silica nanospheres and then composited it with GelMA through UV light crosslinking to form hydrogels [115]. Such a method can provide a stable release of loaded drugs. In addition, Ca^2+^ from HA can create a bridge with the hydroxyl group in GelMA, forming a weak bonding between gelatin and HA [116]. Such composites’ cell viability and biocompatibility are superior, and they are easier for in situ curing simultaneously.

#### 2.4.3. Chitosan

There are various forms of chitosan-based scaffolds in bone tissue engineering, including films, particles, hydrogels, fibers, and sponges [117]. Chitosan is introduced as a linear polysaccharide and has favorable biocompatibility, bioactivity, and biodegradability features. More importantly, chitosan contains free amino groups that can be protonated, making chitosan modifiable with biochemical groups. The protonated amino groups allow the electrostatic interaction with DNA, proteins, lipids, or negatively charged synthetic polymers [118]. A study grafted GRGDSPK (RGD) or FRHRNRKGY (HVP) sequences on chitosan and tested the sample with osteoblasts [119]. The functional groups improved cell adhesion and proliferation. Despite this, the main drawback of chitosan is their low mechanical strength for load-bearing defects. Making a composite with mechanical enhancers is usually applied to overcome this limitation. For example, a PCL fibrous scaffold was introduced for the inclusion of chitosan nanoparticles for a rat model of the critical-sized calvarial bone defect [120]. The hydrophilic nature of chitosan reduced the hydrophobic nature of PCL nanofibers. The presence of chitosan also regulated cellular functions by increasing protein adsorption, fluid uptake, and ALP activity. In another study, the incorporation of bioceramic into the chitosan matrix was evaluated [121]. The histopathological and microbiological results of the composite in an osteomyelitis animal model revealed the ability of chitosan and the calcium phosphate scaffold to induce cellular differentiation and augment the osteoconductive and mechanical properties. The superiority of modification and antibacterial properties make chitosan an excellent choice for functional bone implants, while the suitable mechanical properties demand a prompt solution.

#### 2.4.4. Alginate

Alginate, a natural and anionic polysaccharide, has a great potential for bone tissue engineering due to its biocompatibility, gel-forming ability, and modifying capacity [31]. The studies of alginate scaffolds focus on improving biodegradability, strength, gelation property, and cell affinity. Recently, palygorskite, bioactive glass, graphene oxide, and PCL have been used to prepare composites with alginate for bone scaffolds [122,123,124,125]. Developing injectable alginate-based hydrogels with proper adhesivity and osteogenic activity for utilization in filling bone defects and cavities has always been a tempting goal for researchers. Since complex chemical compositions usually cause difficulties in batch productions, developing a binary component multifunctional alginate-based hydrogel for bone regeneration was investigated. First, using an amidation reaction, dopamine (DA) was grafted to alginate. Then, mixing strontium ions with Alg-DA solution resulted in an injectable hydrogel with proper adhesivity due to catechol groups on Alg-DA. In addition, over 8 weeks of in vivo studies on rats, the enhanced osteogenic activity of strontium containing hydrogel scaffolds was indicated compared to hydrogels without strontium [126]. Tunable void-forming alginate-based hydrogels are excellent choices for filling bone cavities. Another study investigated the potential of alginate-based hydrogels containing rat mesenchymal stromal cells for bone regeneration for critical-sized femoral defects in rats. After 6 weeks post-surgery, the bone and tissue mineral density in the defect site that filled with MSCs encapsulated hydrogel were much higher than the non-cell seeded scaffold. However, none of the hydrogels could repair the defects completely [127]. Despite the benefits, the absence of regulated biodegradability can have undesirable consequences. It should be combined with other biodegradable polymers to eliminate this limitation. One instance of these combinations is chitosan–alginate to repair the physical injury in rats. The proposed hydrogel demonstrated significant controllable degradation that would inhibit bone growth deformities, and also it showed the ability for loading chondrogenic factors. Therefore, this scaffold can be a promising platform that improves physical injury repair [128]. To sum up, alginate has excellent biocompatibility and devisable potential with its functional groups; the limitations such as the strength and degradation of alginate are still the research priorities in this field.

#### 2.4.5. Silk Fibroin

Compared to other natural polymers, silk fibroin (SF) possesses several significant advantages such as excellent biocompatibility, outstanding mechanical properties, and biodegradability [129]. The fibrous structure is the typical characteristic of SF. SF scaffolds with low porosity and thinner fibers can inhibit the immune activation of macrophages and T cells. Yang et al. fabricated an SF-based scaffold with different porosity and fiber thickness through electrospinning [130] and confirmed that the inflammatory response could be regulated through different silk fibroin architectures.

Functionality for biomedicine has been one of the research focuses for SF. Recently, the literature aimed to investigate SF’s cell adhesion, drug-loading capacity, and osteoinductivity [131,132,133,134,135]. Some materials are usually applied to composite SF in hard tissue engineering to improve the biological properties. For example, HA is frequently used to coordinate SF for bone tissue scaffolds. The durability of silk fibroin can precisely make up for the shortcoming of HA to form a scaffold with the ideal mechanical properties. The HA-SF slurry demonstrated shear thinning behavior characteristics, making flow-based injection more clinically convenient [136]. The mechanical study showed that injection and compression molding could provide favorable strength for SF-based scaffolds. A compatible combination between SF and HA has been studied in hard tissue engineering [137]. Similar to HA, bioactive glasses (BGs) are also suitable for mixing with SF to improve biocompatibility and osteoconductivity. In a study, a composite scaffold comprised of SF/BG was constructed by the 3D printing fabrication technique. Bone marrow stem cells were seeded before transplanting into the back of nude mice [138]. The osteogenic ability of the scaffolds was confirmed with enhanced osteogenesis-related genes (COL-1, OCN, BSP, and BMP-2) expression. Synthetic polymers are also applied with SF for fiber scaffolds. An SF-coated PCL scaffold developed by Xiao et al. could improve tissue arrangement and remodeling and support a faster regeneration rate in the rat model [139]. The scaffold’s porosity with electrospinning and gas-foaming technology was much higher than traditional nanofiber mats.

The summary of natural polymer based materials for bone regeneration shown in Table 2.

## 3. Skeletal Muscle

The skeletal muscle connects to the bones by tendons and forms nearly 40% of the total body mass. Skeletal muscles play a significant role in skeletal support and movement, regulation of metabolism, and temperature. Muscle fibers are composed of many myofibrils, and myofibrils contain many myofilaments. Myofibrils are arranged in a unique pattern to form sarcomeres [160], which is the basic contraction unit of skeletal muscle. The two most essential filaments are actin and myosin, which are arranged uniquely to form various bands on skeletal muscle. Skeletal muscle consists of multinucleated single muscle cells called myofibers. Muscle stem cells are distributed at the periphery of the myofibers, making up 1 to 5% of total muscle cells [161]. These cells multiply in response to mechanical and chemical damage and cause growth, replacement, and repair of the tissue [162,163,164,165,166,167]. Skeletal muscles are joined to the nervous system for activation and contraction and the blood vessels for the diffusion of nutrients and oxygen and waste effusion.

### 3.1. Skeletal Muscle ECM Structure

**Figure 3:**
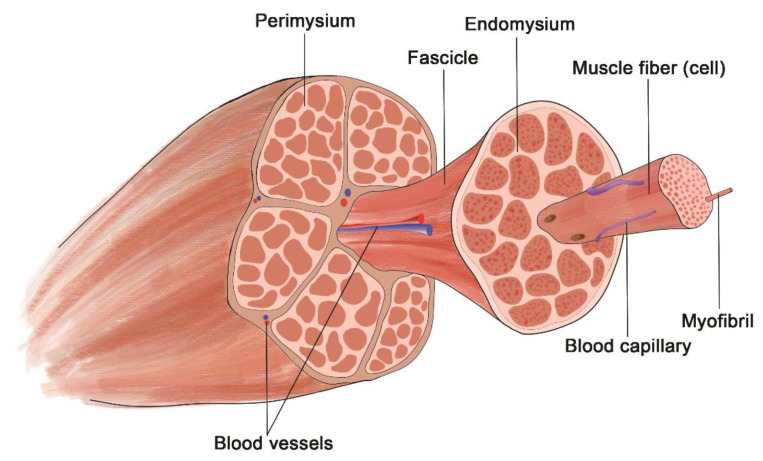
Structure of Skeletal Muscle.

Skeletal muscle tissue’s extracellular matrix (ECM) is complex with a highly organized structure [168]. The ECM plays a vital role in the growth, development, repair, muscle elasticity, regeneration, cell function, and force transmission in the muscle [169]. In addition to mechanical support for cells, the ECM also plays a host of signaling cascades [166]. The main components of the ECM structure are collagen, glycoproteins, proteoglycans, and elastin. The most abundant collagen types in skeletal muscle tissue are collagen type I and III [170]. Skeletal muscle tissue has two separate parts of the ECM structure: the basal lamina, which has a sheet-like structure, and intramuscular connective tissue, with an organized structure consisting of three major parts, as shown in Figure 3 described below [166,171].

The muscle is composed of myocytes arranged in bundles. The length of each cell varies, and the cells are closely spaced and complementary in length. Each cell is wrapped with a thin reticular membrane, which is called the endomysium; each muscle bundle is enfolded with a connective tissue membrane mixed with glial and elastic fibers, which is called the fascicle membrane; outside of each muscle, there is a thicker layer of connective tissue, which is called the epimysium. The connective tissues of each membrane are continuous, and the blood vessels and nerves distributed to the muscles enter along the connective tissue membrane [165,166,168].

### 3.2. Disorders

Injuries and disorders such as traumatic injuries, surgical procedures, and congenital and acquired diseases that result in complete and irrecoverable loss of skeletal muscle function have been known as volume muscle loss (VML). The standard VML treatment is autologous transplantation of skeletal muscle from a cadaver or a donor. However, this approach is costly and time-consuming, and it is associated with immune response and donor site morbidity. Tissue engineering approaches have been developed as an alternative to overcome these complications. Many scaffolds combined with cells, drugs, small molecules, or growth factors have been used in tissue engineering applications [172,173,174].

### 3.3. Natural-Based Scaffold for Skeletal Muscle Tissue Engineering

#### 3.3.1. Keratin

Keratin is known as a carrier for the primary fibroblasts growth factor (bFGF or FGF-2), insulin-like growth factor 1 (IGF-1), and vascular endothelial growth factor (VEGF) [172]. bFGF directly regenerates muscles by enhancing the proliferation of the satellite cells. Similarly, IGF-1 plays an essential role in muscle maintenance and regeneration. VEGF is a protein that plays a positive effective role in angiogenesis, which increases the longevity of tissue-engineered skeletal muscle [172,173,174]. Keratin contains growth factors that significantly elevate the formation of new muscle tissue, myofibers, and blood vessels and reduce fibrosis [173]. The binding between those cytokines and keratin can prevent rapid degradation and achieve controlled release [175]. The in vivo implantation of keratin hydrogel in combination with IGF-1, bFGF, or muscle progenitor cell (MPCs) as a scaffold in rat tibialis anterior muscle VML injury model demonstrated significant improvement in the regeneration of skeletal muscle tissue. In another in vivo study, the scaffold and MPCs, VEGF, IGF-1, and bFGF were examined. This study proved a diminished inflammatory response and an enhanced muscle re-formation [174]. These studies suggested that keratin hydrogel, along with growth factors, improves treatment performance in VML injury.

Keratin is also frequently mixed with synthetic polymers, especially PCL [176,177]. Commonly, the keratin composite scaffolds for muscle tissue are prepared by electrospinning. Keratin contains a large amount of nitrogen that produces NO, which is one of the metabolism products of keratin [177]. Due to the catalytic generation of NO, the PCL/keratin composite scaffold can accelerate endothelial cell growth and reduce smooth muscle cell proliferation [178]. Such keratin-based scaffolds are a NO donor in the blood, benefiting vascular tissue regeneration.

#### 3.3.2. Collagen

Collagen is the central part of ECM, which increases the formation of new blood vessels and muscular tissue [179]. The application of murine muscle-derived stem cells (MDSCs) and collagen for the regeneration of muscle defects has been reported. The results demonstrated better skeletal muscle regeneration, higher cell proliferation, and reduction in fibrotic scar formation in the collagen scaffolds with MDSCs compared to only collagen scaffolds [180]. In an in vivo study, a mice VML injury model was used to screen different scaffolds. It was reported that collagen type I and an ECM hydrogel demonstrated better cell viability and VML treatment. The following indicated that the ECM-based scaffold (in comparison with the collagen type I hydrogel) led to the highest number of myofibers [181].

Collagen and glycosaminoglycan (GAG) (chondroitin 6-sulfate) were applied as a scaffold to regenerate a mice VML injury model. Chondroitin sulfate is one of the most critical components in cartilage structure and plays a vital role in the formation of skeletal muscle tissue and the regeneration of muscular tissue [179]. The collagen–GAG scaffold led to elevated expression levels of growth factors related to muscle tissue. A mice VML model treated with the scaffold also showed a reduction in fibrosis compared to untreated VML [179]. The research on collagen composites for muscle tissue engineering often contains synthetic materials. PCL, polypyrrole (PPy), and polyvinyl alcohol (PVA) have been recently used with collagen for skeletal muscle tissue engineering [182,183,184]. These combinations can reinforce collagen and provide various functions for the scaffolds. For instance, collagen mixed with conductive PPy nanoparticles promoted cell adhesion, growth, and proliferation [182]. Furthermore, enhanced myotube formation and maturation were found in another collagen/PPy implantation study [185].

Muscle is well-aligned tissue with fibrous structures at various levels. Therefore, the scaffolds for skeletal muscle tissue should be aligned. A murine model used collagen-aligned scaffolds comprising mouse myoblast and human microvascular endothelial cells to treat VML injury. The results indicated that collagen-aligned nanofibrillar scaffolds promote the regeneration of skeletal muscle and angiogenesis in comparison with randomly oriented ones [186]. Lotus-root-like collagen scaffolds prepared by Hwangbo et al. showed a more bio-stimulating structure than conventional collagen struts [183]. The aligned hierarchical microtubular collagen niche can enhance cell adhesion and promote myogenic differentiation and maturation. Such a porous structure is also necessary for angiogenesis in soft tissue regeneration.

#### 3.3.3. Alginate

This abundant biopolymer is not only biocompatible and has low toxicity but also exhibits a temperature-independent gelation process in the presence of divalent cations, making it an excellent candidate for tissue engineering [187]. The partial oxidation of alginate is a common way of controlling biodegradability, and it is mainly used for tissue regeneration purposes [188]. The wet-spun fabrication of alginate fibers containing muscle precursor cells is reported to be efficient for muscle recovery based on an in vivo study on a mouse model [187]. Another work used an injectable 3D RGD-coupled alginate scaffold to deliver gingival mesenchymal stem cells for muscle regeneration and confirmed effective muscle regeneration in mice [189]. Oxidized alginate-gelatin bioink was also used for 3D printing of mouse myoblast cells (C2C12). The results showed that the proper selection of nozzle size extrusion pressure could affect cell orientation and migration in the printed scaffold for muscle regeneration [190]. A new approach was also reported for muscle regeneration exploiting the interplay between specific cell membrane receptors. This research utilized borax-loaded alginate hydrogels to stimulate the borate transporter, NaBC1. In vivo studies of this approach on mice showed a successful acceleration of the muscle regeneration process [190].

#### 3.3.4. Laminin, Fibrin, and Gelatin

Laminins are heterotrimeric glycoproteins that are naturally formed by the muscle and localized in ECM consequently. A new hydrogel consisting of fibrinogen and laminin-111 (laminin-111 enriched with fibrin) was applied to treat a murine model of VML injury. The different properties of laminin trimers allow cell receptors to regulate different cellular pathways [191]. The LM-111 scaffold significantly improved muscle weight and increased the penetration of satellite, endothelial, hematopoietic, and immune cells [192]. Adipose-derived stem cells (ASCs) can be used in muscle tissue engineering applications. The combination of ASCs and electrospun fibrin fibers can mimic the native tissue. Following in vivo implantation, the ASCs seeded on a fibrin scaffold did not significantly enhance muscle regeneration [193].

In situ bioprinting of GelMA hydrogel was employed to treat VML injury. The use of encapsulated cells in this study led to the formation of multinucleated myotubes [194]. The most attractive part of this study is the direct-printing technology used in the defect area. In situ crosslinking allows surgeons to fill VLM injury rapidly and adequately, significantly improving tissue regeneration and functional recovery.

Recently, Hwangbo et al. used an in situ UV crosslinking hydrogel to treat VML by two different bio-inks, GelMa and C2C12 or GelMa and human adipose-derived stem cells (hASCs). They optimized printer parameters such as barrel temperature, number of UV light sources, UV exposure dose, and wall shear stresses at the first step. Next, bio-printed structures laden with hASCs were implanted into mice as in vivo tests and showed a significant improvement in muscle regeneration. Based on the reported result, they developed a promising in situ crosslink GelMa construct for treating VML [195].

Natural polymers alone are not suitable for treating injuries such as VML due to their poor mechanical properties. Thus, combining hydrogels, growth factors, and cells increases skeletal muscle regeneration.

The summary of natural polymer based biomaterials for skeletal muscle regeneration is shown in Table 3.

## 4. Tendon and Ligament

Despite having essential and unique functions in the musculoskeletal system, research on tendons and ligaments is not as advanced as the rest of skeletal tissues [219]. Tendons and ligaments are very similar but still distinct connective tissues. According to this, tendons are fibrous tissues that join skeletal muscle to bone, making movements possible through force transmission from muscles to bones [220]. At the same time, ligaments are the dense fibrous connective tissue that connects bone to bone [221]. The transmission of these tensile forces by tendons and ligaments makes them susceptible to tearing or complete rupture, depending on the amount of the force [222].

The use of natural and synthetic polymers for tendon and ligament tissue engineering has been investigated for years, and obviously, each has its pros and cons. For example, better cell attachment to synthetic scaffolds with dense, fine, and aligned fibers and a tendon-like cellular phenotype on synthetic scaffolds have been reported. On the other hand, biological scaffolds promote better cell proliferation and the expression of collagen genes, the most abundant molecular component in tendon and ligament [223]. Hence, using both natural and synthetic polymers to maintain both biological and mechanical requirements simultaneously seems logical.

### 4.1. Tendon and Ligament ECM Structure

**Figure 4:**
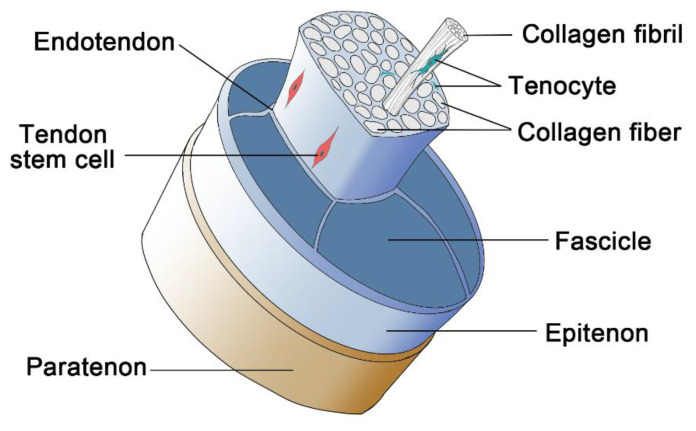
The hierarchical structure of tendon and ligament.

Tendons and ligaments have very similar ECM components and structures. At the microscale, they both have wave-form patterns with fibers oriented parallel to the stress axis. They are straightened when put under tension and reconverted when released. They both have a hierarchical structure, beginning with collagen molecules, fibrils, fiber bundles, fascicles (considered the basic functional unit of the tissue), and ultimately tendon and ligament units [224,225] (Figure 4). The tensile strength of the tendon is reported to be about 50–150 MPa, and its elastic modulus is about 1200–1800 MPa, while the ligament has a tensile strength of about 50 MPa and elastic modulus of about 150–355 MPa. They both have a certain degree of plasticity for adaption to changing stresses [225]. They both follow the elastic model up to a certain amount of strain. Afterward, they will undergo microscopic failure, and further strain may lead to a total rupture of the tissue [226].

The chemical composition of the tendon and ligament is very similar, with a slight difference in the number of components. The main component of both tendon and ligament is water, 60% to 80% in weight [220,221]. They contain a protein phase (collagen) and a polysaccharide phase (proteoglycans). Collagen type I is the most abundant protein in the tendon and ligament [225]. It constitutes about 60% of the tendon’s dry weight and corresponds to 95% of the total tendon collagen. The other 5% involves mainly collagen types III and V. There are minimal amounts of collagen types II, VI, IX, X, and XI. On the other hand, ligaments contain more protein, less total collagen, and greater amounts of type III collagen and GAGs [221]. While many of these collagen types’ full biological and biophysical roles are still unclear, some specific functions of each type have been identified [224]. Elastin is another important component of both tendon and ligament, which is responsible for recovering the native configuration after stretching [226]. The proteoglycans found in tendons and ligaments, including decorin, aggrecan, tenascin C, fibronectin, fibromodulin, biglycan, and lumican, have specific functions mainly to organize and lubricate collagen fiber bundles [219,226].

Tenoblasts and tenocytes are the two main cell types present in tendons. Tenoblasts are very active spindle-shaped immature tendon cells that can be found as clusters in some areas of tendons. They are the predominant cell type in the tendon that can mature into tenocytes with fibroblastic morphology and low metabolic activity. Other types of cells present in the tendon are progenitor cells, synovial cells, endothelial cells, and even chondrocytes [221]. The primary cell type of ligament is fibroblasts, and these cells help in the production of collagen and matrix remodeling by the degradation of the pre-existing collagen [225].

### 4.2. Disorders

Lesions of tendon and ligament account for over 40% of musculoskeletal injuries [227]. These injuries are widespread in the elderly and very physically active persons such as athletes. Half (50%) of all sports injuries are related to lesions of tendons and ligaments [228]. Two of the most common ligaments exposed to the risk of injury are the Anterior Cruciate Ligament (ACL) and Deltoid Ligament (DL) of the ankle. Ankle sprains or sports accidents are the leading cause of injury to ACL and DL [229,230]. The most common tendons exposed to the risk of injury are the Achilles tendon, Flexor/Extensor tendons of the hand, and the rotator cuff shoulder tendons [231]. The hypocellularity and hypervascularity of these tissues reduce their natural intrinsic healing ability. Thus, full recovery is relatively difficult [232]. The healing process follows three typical steps: inflammatory, proliferative, and remodeling phase. The latter is characterized by the alignment of collagen fibers parallel to the axis of muscle force direction, which plays an important role in the recovery of biomechanical properties of the tissue. Natural healing typically forms scar-like tissue with poor biomechanical properties that cannot have the proper functionality. The most common mode of surgical repair for these injuries involves using different suture techniques for reattachment. However, this method’s high chance of failure and re-rupture provides excellent room for improvement. Crosslinking agents, bio-patch, or grafts to cover the ruptured area and the sutures for strengthening the repair of ruptured connective tissue have been proposed to overcome the failure and re-rupture [233]. The gold standard for surgical procedures is autografts, which have several limitations. As alternative commercialized allografts and xenografts are available, these have the risk of rejection and disease transmission. None of these approaches is considered the best [234]. However, using these tissue engineering techniques is necessary because an aberrant natural wound healing would result in excessive collagen synthesis and the formation of scar-like tissue (fibrosis) with poor biomechanical functionality [235]. Therefore, a sound understanding of the production and assembly of type I collagen fibrils is fundamental for tendon and ligament biomaterials engineering [236].

### 4.3. Natural-Based Scaffold for Tendon and Ligament Tissue Engineering

Despite all the valuable research in tissue engineering for tendons and ligaments in recent years, there are still many material and method selection challenges. Multiple factors should be considered when developing new therapies: on the one hand, perfect biocompatibility, proper biodegradability, and the ability to mimic the native ECM of the targeted tissue. On the other hand, having good functionality and biomechanical properties have made it difficult for researchers to agree on one biopolymer. Recently, collagen and silk have attracted much interest in this research area.

#### 4.3.1. Collagen

The first material that has been considered for tendon and ligament implants is collagen type I, as it is the most abundant polymer in the structure of the tendon and ligament. However, natural polymers alone usually lack the required strength and biomechanical properties. A common way to increase the mechanical properties of natural polymers is to use them along with synthetic polymers. In 2021, the use of hybrid material of poly-L-lactic acid (PLLA)-based copolymers with collage/chondroitin sulfate was investigated [236]. After implantation in rats, it was observed that the collagen/chondroitin sulfate/PLLA rod enhanced cell proliferation and in vivo collagen fibrillation, suggesting benefits for tendon regeneration. In another study, electrospun PCL fiber was composited with collagen to fabricate the ligament scaffolds. With its outstanding elasticity, PCL is a perfect match with natural polymer for tendon and ligament repair [236]. The porous core-shell scaffolds were also doped with proteoglycans and glycosaminoglycans (GAG). Both are essential components of ECM, allowing cells a more appropriate space for migration. At the same time, growth factors can be applied to improve the performance of collagen scaffolds. One recent study confirmed that collagen sponge scaffolds with TGF-β1 and GDF-7 can promote tenogenic differentiation [237].

#### 4.3.2. Silk

Silk attracts much interest from researchers to fabricate artificial scaffolds for tendons. As a linear material, the mechanical properties of silk fibroin are anisotropy, leading to the possibility of anisotropic functionalization. Chen et al. fabricated a gradient bio-mineralized silk fibroin nanofibrous scaffold [238]. The combination of silk fibroin and synthetic polymer has also been investigated. For example, nano-yarn scaffolds made of PLLA/PCL/silk fibroin for ACL reconstruction in rabbit were reported [239]. In this study, both sufficient cellularity and higher modulus and stiffness are reported after 12 weeks of implantation compared to the control group due to collagen and silk.

In recent years, there have also been other studies using other natural biopolymers such as chitosan, alginate, cellulose, and fibrin [240,241,242,243,244]. Hybrid natural polymers like alginate–chitin scaffold that improved supraspinatus tendon-to-bone healing in vivo is also reported [245]. These studies are summarized in Table 4.

## 5. Conclusions

Current natural polymer scaffold research for MS tissue engineering focuses on improving existing materials and preparation processes and exploring novel naturally inspired materials. The number of studies using only one natural polymer for MS tissue engineering is declining in recent years, while compositing different materials together is becoming more popular. A single polymer cannot satisfy all the expectations and requirements for a perfect scaffold for MS tissue engineering purposes. However, a combination of polymers may contribute to the structure’s cellular and mechanical aspects. An ideal strategy is to take advantage of each type of material and combine them. The development of natural polymer scaffolds has become a hot spot in the research field. Moreover, it has shown excellent application prospects, giving various possibilities for developing artificial organs, injury repair, and disease treatments. It will promote tissue engineering research forward to a mature stage.

Considering recent research, natural polymers have many merits as implantable materials for MS tissue engineering. However, natural polymer-based implants have specific issues that need to be addressed: (1) Natural source polymers are uncontrollable from the initial production phase. As a result, each batch of natural biomaterials might have a varied quality. Therefore, it is necessary to harmonize the quality standards of materials, strictly control their quality, and strengthen the research of fundamental theories such as the properties and structure of materials. (2) There is a contradiction in mechanical properties, degradation speed, and permeability between biopolymers. Polymers with high molecular weight or stable structure usually have higher strength, and their degradation speed and permeability are challenging to meet the requirements in tissue engineering, especially in in vivo and clinical studies. Advanced strategies to break through this barrier are promising in polymer science (3) The development of composited biomaterials to meet the requirements of different tissues works very well. However, more research should be completed with different compositing methods instead of simple mechanical mixing. The more complex chemical and/or physical structures would enable accomplishing the regeneration mission compared with a widely used homogeneous structure. (4) The adhesion of cells on natural polymers needs to be further studied. Physically speaking, topology, hydrophilicity, nano/micro pattern, macromolecular structure adjusting, and other polymer characteristics can cater to the cells’ requirements. From a biological point of view, the superior biocompatibility of natural-origin polymers makes them stand out among other materials. Taking natural polymers as one of the first considerations when facing the challenge of inflammation is reasonable. Chemically speaking, the ability of grafting is the most significant advantage of natural biopolymers and should attract widespread attention. The functional groups, such as carboxyl, hydroxyl, and amidogen, provide potential for design, modification, and functionalization. Apart from this, the metabolites of degraded polymers might have a functional contribution to tissue regeneration and have not been researched extensively. Other study methods such as drug or micro/macro-molecular doping are also the research focuses. In addition to these obstacles, existing natural biological materials, including their derivatives, have tremendous potential for further research and development, such as the chemical modification of chitin and hyaluronic acid to produce a variety of derivatives, making it more suitable as a scaffold material in musculoskeletal tissue for clinical application.

## Figures and Tables

**Table 1:** Characterizations of the natural polymers used in tissue engineering.

Materials	Structure	Sources	Key Features	Ref
**Chitosan**	Linear polysaccharide	The shell of crustaceans (crabs, lobsters, shrimps, crayfish, and king crabs) as well as mollusks (e.g., squids), cuticles of insects, and cell walls of fungi	Second most abundant natural polymer, Biocompatible, Biodegradable, Bioadhesive, Biologically renewable, Antimicrobial, Hemostatic nature, Non-antigenic, Antioxidant, pH-sensitive	[27,28,29]
**Alginate**	Linear polysaccharide	Seaweeds and typically extracted from brown algae	Biocompatible, Biodegradable, Cytocompatible, Non-immunogenic, Mucoadhesive, Source abundance, Low cost, Water-soluble, pH-sensitive, in situ gelation	[30,31,32,33]
**Starch**	Composed of two kinds of polysaccharides, amylose, and amylopectin	The leaves of all green plants and in the seeds, fruits, stems, roots, and tubers of most plants and also in algae	Biocompatible, Biodegradable, Biorenewable, Low cost, Semicrystalline, High mechanical strength	[34,35,36]
**Hyaluronic acid**	Linear polysaccharide	A major macromolecular component of the ECM in the most connective tissues	Biocompatible, Biodegradable, Bioresorbable, Limited immunogenicity, Recognized by cell surface receptors, Flexible, Unique viscoelasticity	[37,38,39,40]
**Chondroitin** **sulfate**	Unbranched polysaccharide	A major component of ECM	Biocompatible, Biodegradable, Easily available, Immune-enhancing activity, Anti-inflammatory, Antioxidant, Antitumor, Anti-coagulation	[41]
**Agarose**	Liner polysaccharide	Marine red algae and also found as a support structure of cell wall for marine algae	Biocompatible, Non-immunogenic, Water solubility, pH-sensitive, Electro-responsive activity, Thermoreversible gelation behavior	[42,43]
**Bacterial Cellulose**	Linear polysaccharide	Microorganisms belonging to the Gluconacetobacter xylinum	Biocompatible, Biodegradable, High water-holding capacity, High mechanical strength, Porous structure, High crystallinity	[44,45,46,47,48]
**Dextran**	Branched polysaccharide	Lactic-acid bacteria	Biocompatible, Low cost, Easy to modify, Stable under mild acidic/basic conditions, Slowly degraded	[49,50,51]
**Carrageenans**	Linear polysaccharide	Marine red algae	Viscoelastic and gelling properties, Anti-inflammatory, Antitumor	[52]
**Gellan gum**	Linear polysaccharide	Sphingomonas elodea or Pseudomonas elodea bacteria	Minimal cytotoxicity, Ability to form hard and translucent gels which are stable at low pH, Thermally reversible gel in the presence of metallic ions	[53,54,55]
**Xanthan gum**	Branched polysaccharide	Xanthomonas bacteria	Biocompatible, Non-toxicity, Biodegradable, Stabile under a broad spectrum of pH, Shear-thinning	[56]
**Heparin**	Linear polysaccharide	Mucosal tissues such as the porcine intestine or bovine lungs	Antitumor, Anti-viral, Angiogenesis regulatory activities	[57,58]
**Collagen**	Fibrous protein	A major ECM component of most connective tissues within the mammalian body	Biocompatible, Biodegradable, Low-immunogenic, Hemostatic, High swelling ability, Low antigenicity, Capacity to facilitate cellular attachment	[59,60,61,62]
**Gelatin**	Protein	A hydrolysis derivative of collagen	Biocompatible, Biodegradable, Non-immunogenic, Elastic, Lower antigenicity, More accessible functional groups	[63,64,65,66]
**Silk fibroin**	Protein	Silkworms and spiders	Biocompatible, Biodegradable, Great mechanical properties, Versatile processability	[67,68,69,70,71]
**Keratin**	Polypeptide	A major component in nail, skin, hair, horns hooves, wool, feathers	Biocompatible, Biodegradable, Possesses cellular interaction sites Low-immunogenic, Intrinsic ability to self-assemble into three-dimensional structures	[72,73,74,75]
**Fibrin**	Glycoprotein	Fibrinogen	Biocompatible, Biodegradable, Ability of monomers to self-assemble into a gel	[76,77,78,79]
**Elastin**	Structural protein	A component in the ECMs of connective tissues (e.g., blood vessels, esophagus, skin)	Biocompatible, Biodegradable, Elasticity, Self-assembly, Long-term stability	[80,81]

**Table 2:** Summary of recent studies using natural polymers in bone tissue engineering.

Ref	Applied Materials	Cell Type	Structure/Production Method	Benefits
[140]	HA/gelatin/chitosan	Human osteoblast-like cell line (MG-63)	Core–shell nanofibers/freeze-drying method and calcium ion crosslinking	Biomimetic porous 3D scaffold with gradient and layered microstructure
[141]	Gelatin–alginate graphene oxide	Human osteoblast-like cell line (MG-63)	Nanocomposite scaffold/freeze drying technique	Enhanced compressive strength, 700% swelling ratio, slow biodegradation (≈30% in 28 days)
[142]	Gelatin-bioactive glass-ceramic	Human osteoblast-like cell line (MG-63)	Macroporous composite/lyophilization	Controlled degradation of gelatin scaffold and enhanced mechanical strength by incorporation of bioactive glass particles
[143]	Carboxymethyl chitosan/PCL	Human osteoblast-like cell line (MG-63)	Nanofibrous scaffold/electrospinning	Ultrafine and splitting fibers, reduced water contact angle
[144]	Chitosan/honeycomb porous carbon/HA	Bone marrow mesenchymal stem cells	Hierarchical porous structures/vacuum freeze-dried	Suitable pore size and high porosity for cell viability, mineralization, proliferation, and osteoinduction
[145]	Alginate/chitosan-HA	Human chondrocytes and fibroblasts	Porous gradient scaffold/freeze-drying and crosslinking by calcium ions	High compression modules and porosity
[146]	Gelatin/alginate/polyvinyl alcohol	MC3T3-E1 pre-osteoblast cells	Macroporous 3D spongy scaffold/cryogelation technique	Anti-bacterial scaffold for bone regeneration
[147]	Gelatin	L-929 fibroblasts, D1 MSC and MG63 osteoblasts	Fiber scaffold/freeze-dried	Enzymatically crosslinked scaffold for bone regeneration
[148]	Gelatin/PLLA	L929 fibroblasts	Multifunctional layered scaffold/electrospinning and 3D printing	Nasal cartilages and subchondral bone reconstruction
[149]	Strontium-Substituted HA/Gelatin	Coculture of osteoblasts and osteoclasts	Porous 3D scaffold/freeze-drying	Useful for local delivery of strontium and excessive bone resorption ability
[150]	Gelatin/PCL/nanoHA/vitamin D3	Human adipose-derived stem cells	Nanocomposite scaffold/electrospinning	nHA and vitamin D3 have a synergistic effect on the osteogenic differentiation of hADSCs
[151]	Collagen/silica	Lymphocytes	Collagen fibrils with deposition of intrafibrillar amorphous silica	Promoting bone regeneration and angiogenesis via monocyte immunomodulation. Differentiation of blood-derived monocytes into TRAP-positive cells due to sustained release of silicic acid
[152]	Fibroin/poly(lactide-co-ε-caprolactone)	Human adipose-derived stem cells	Hybrid nanofibrous scaffold	Inducing cell adhesion and proliferation, favorable tensile strength, and surface roughness
[153]	Fibroin/PLGA	Rat bone marrow mesenchymal stem cells	Core–shell nanofibers	Enhancing cell adhesion, diffusion, and proliferation, promoting the osteogenic differentiation
[154]	SF/cellulose/chitosan	Human osteoblast cell line	Composite Porous scaffold	Supporting cell proliferation and promoting biomineralization
[155]	Fibroin/gelatin	Rat mesenchymal stem cell	Composite microcarrier	Supporting cell adhesion, proliferation, and elastic modulus
[156]	Alginate/nano-HA	Rat calvaria osteoblast	Composites	Good bioactivity, high biocompatibility, antibacterial activity
[157]	Silk/calcium silicate/sodium alginate	Bone marrow stromal cells	Hydrogel	Good biodegradation, cytocompatibility, bioactivity, and the proliferation of bone marrow stromal cells
[158]	Alginate/calcium phosphate paste	Stem cells	Injectable microbeads	Enhancing cell viability, proliferation, osteogenic differentiation, and bone regeneration
[159]	Alginate/gelatin/apatite coating	Rat bone marrow stem cells	3D printed composite scaffold	Higher proliferation, osteogenic differentiation, surface protein adsorption, and Young’s modulus for apatite-coated scaffold

**Table 3:** Summary of recent studies using natural polymers in skeletal muscle tissue engineering.

Ref	Applied Materials	Cell Type	Structure/Production Method	Advantages
[196]	Collagen/PPy	C2C12 mouse myoblast	3D, highly aligned, and electrically conductive collagen scaffold via directional lyophilization of a polypyrrole-doped collagen suspension	Increasing electrical conductivity by using polypyrrole (PPy)
[197]	Collagen	C2C12 murine skeletal muscle myoblast cell	Fused deposition modeling (FDM)	Increased IGF1 mRNA and, Akt, p70S6K, and 4EBP1 phosphorylation, along with myotube hypertrophy and improved designed muscle functionality
[198]	Alginate/Gelatin/Heparin	Human skeletal muscle progenitor cells (hSMPCs)	Hydrogel	Cost-effective and an alternative for commercial biomaterials
[199]	Alginate	Mesenchymal stromal cells (MSCs)	Hydrogel	IGF-1 and VEGF165 had significant effects on muscle progenitor cells
[188]	Alginate/Gelatin	C2C12	Extrusion-bioprinting of hydrogel	Alginate–gelatin hydrogel is a simple and cost-efficient biodegradable bio-ink
[200]	Gelatin/Hyaluronic acid	C2C12	Hydrogel	Myotube production was established throughout the hydrogel when both gelatin and hyaluronic acid were present, and no shrinkage occurred
[201]	Fibrin/Polyethylene oxide (PEO)	C2C12	C2C12s are encapsulated and electrospun into fibrin/polyethylene oxide (PEO) microfiber bundles with aqueous solution electrospinning.	Loading C2C12s as cellular aggregates increasing cell viability
[202]	Fibrin	Muscle progenitor cells (MPCs) adipogenic	Hydrogel	Adipogenic differentiation was decreased by myogenic differentiation but not prevented, and MPCs produced from diabetic animals had a higher capacity for adipogenic differentiation.
[203]	Fibrin/Laminin	C2C12	Hydrogel	Integrating laminin-111 into fibrin hydrogels is possible
[204]	Fibrin/Alginate	C2C12	Three-dimensional engineering of skeletal muscle tissue using electrospun fibrin microfiber bundles	To promote tissue formation, myoblasts should undergo biophysical stimulation
[205]	Fibrin/Thrombin	C2C12	3D printing, co-extruding fibrinogen and thrombin	Enhancing the regeneration of functional muscle tissue by tuning the topographic features of scaffolds
[206]	Fibrin/Collagen	Primary human skeletal muscle cells	Hydrogels	The Young’s modulus increased twofold, maximum strain decreased 2.5 times, and collagen deposition increased 1.6 times
[207]	Gelatin methacrylate (GelMA)	C2C12	Under single UV exposure, silicone tubes-based coagulant produces cell-laden GelMA microfibers	Increased uniaxial strain ratio of up to 35–45% and significantly improved myotube contractility
[208]	Fibrin + Alginate	Primary human myoblasts	Injectable gel	Optimization of myoblast transplantation can include consideration of cell state
[209]	Fibrin/Alginate/Collagen	Human umbilical vein endothelial cells (HUVEC)	The use of 3D printing to create scaffolds composed of multiple gel layers and hollow channels	They developed a very cost-effective 3D printing system
[210]	Fibrin/Collagen-I	Mesenchymal stem cells (MSCs)	Parallel nanofiber electrospinning	When myogenic differentiation occurs, IGFBPs play a role, varying based on culture and stimulation conditions.
[211]	Fibrin	Muscle-derived stem cells (MDSCs)	Gel	SW033291 increased MDSC myogenic differentiation and myotube creation in a significant way.
[212]	Gelatin	C2C12	Cell-based 3D bioprinting	The dECM components accelerated myogenic differentiation, while topographical cues caused cellular alignment
[213]	Gelatin	C2C12	Cryogel	Myoblasts organize themselves around this pore structure and colonize the entire three-dimensional structure
[214]	Gelatin/Chitosan	L929 fibroblasts cell line	Hydrogel–3D printing	Increased cell viability
[215]	Gelatin/Alginate	C2C12	Hydrogel–3D printing	Adding calcium peroxide (CPO) as an oxygen-generating source to bio-ink can improve cell metabolic activity in Gelma bio-ink
[216]	Gelatin	C2C12	Hydrogel	Soft substrates can support longer-term cell culture
[217]	Fibrin	Bovine satellite cells (BSCs)	Hydrogel	Up to a 15-fold increase in myoglobin expression in vascular smooth muscle cells
[218]	Gelatin	C2C12	Hydrogel	An increase in sarcomere formation in myotube cultures using micropatterned gelatin hydrogels

**Table 4:** Summary of recent studies using natural polymers in tendon/ligament tissue engineering.

Ref.	Applied Materials	Cell Type	Structure/Production Method	Advantages
[241]	Silk/Collagen Polyurethane	L929 fibroblast cell line	Knitted silk covered by electrospun collagen/polyurethane	___
[240]	Collagen/Silk	Tendon stem progenitor cells (TSPCs)	Knitted silk scaffold dipped in collagen solution (in vivo study)	Macroporous structure
[242]	Alginate/Polyacrylamide Silica Microparticles	____	Hydrogel scaffolds dried under stretch	Scaffold production under tension
[243]	Alginate/Cellulose	____	Aligned fibrous hydrogels dried under stretch	Scaffold production under tension
[244]	Fibrin	Rabbit bone marrow-derived mesenchymal stem cells (BMSCs)	2D and 3D fiber based structures	Use of different growth factors
[245]	Collagen/Nanocarbon fibers	___	Electrospun collagen/nanocarbon fibers	Use of nanocarbon fibers
[246]	Bacterial Cellulose	Human mesenchymal stem cells (hMSCs)	Bacterial cellulose sheets	Use of invaluable bacterial cellulose
[247]	PCL/CHT/CNC (Cellulose Nanocrystals)	Tendon-derived cells and adipose stem cells	Aligned electrospun nanofiber threads, braided and woven scaffolds	Reinforcement of mechanical properties by CNC
[248]	PCL/CHT CNCs	Human tendon-derived cells (hTDCs)	Electrospun nanofibrous scaffolds	Reinforcement of mechanical properties by CNC
[249]	PLLA/Collagen	___	Electrospun fibrous structure	CT scans of fiber to compare the morphology with native tendon
[250]	Collagen/PCL	C2C12 cells	Scaffold production using solvent casting and freeze drying including a subsequent crosslinking	Highly interconnected porous scaffold
[251]	Collagen–GAG	Equine tenocytes	Directional solidification of scaffolds	Investigation of scaffold pore size and crosslinking density

## Data Availability

Not applicable.

## References

[1] Loebel C., Burdick J.A. (2018). Engineering Stem and Stromal Cell Therapies for Musculoskeletal Tissue Repair. Cell Stem Cell.

[2] Zumwalt M., Reddy A.P. (2020). Stem Cells for Treatment of Musculoskeletal Conditions—Orthopaedic/Sports Medicine Applications. Biochim. et Biophys. Acta Mol. Basis Dis..

[3] Bai M., Cai L., Li X., Ye L., Xie J. (2020). Stiffness and Topography of Biomaterials Dictate Cell-Matrix Interaction in Musculoskeletal Cells at the Bio-Interface: A Concise Progress Review. J. Biomed. Mater. Res. Part B Appl. Biomater..

[4] Madrigal J.L., Stilhano R., Silva E.A. (2017). Biomaterial-Guided Gene Delivery for Musculoskeletal Tissue Repair. Tissue Eng. Part B Rev..

[5] Ma D., Wang Y., Dai W. (2018). Silk Fibroin-Based Biomaterials for Musculoskeletal Tissue Engineering. Mater. Sci. Eng. C.

[6] Gonzalez-Fernandez T., Sikorski P., Leach J.K. (2019). Bio-Instructive Materials for Musculoskeletal Regeneration. Acta Biomater..

[7] Narayanan N., Jiang C., Uzunalli G., Thankappan S.K., Laurencin C.T., Deng M. (2016). Polymeric Electrospinning for Musculoskeletal Regenerative Engineering. Regen. Eng. Transl. Med..

[8] Nie X., Wang D.A. (2018). Decellularized Orthopaedic Tissue-Engineered Grafts: Biomaterial Scaffolds Synthesised by Therapeutic Cells. Biomater. Sci..

[9] Ferrigno B., Bordett R., Duraisamy N., Moskow J., Arul M.R., Rudraiah S., Nukavarapu S.P., Vella A.T., Kumbar S.G. (2020). Bioactive Polymeric Materials and Electrical Stimulation Strategies for Musculoskeletal Tissue Repair and Regeneration. Bioact. Mater..

[10] Qazi T.H., Mooney D.J., Pumberger M., Geißler S., Duda G.N. (2015). Biomaterials Based Strategies for Skeletal Muscle Tissue Engineering: Existing Technologies and Future Trends. Biomaterials.

[11] Bayrak E., Yilgor Huri P. (2018). Engineering Musculoskeletal Tissue Interfaces. Front. Mater..

[12] Abalymov A., Parakhonskiy B., Skirtach A.G. (2020). Polymer-and Hybrid-Based Biomaterials for Interstitial, Connective, Vascular, Nerve, Visceral and Musculoskeletal Tissue Engineering. Polymers.

[13] Jiménez M., Abradelo C., San Román J., Rojo L. (2019). Bibliographic Review on the State of the Art of Strontium and Zinc Based Regenerative Therapies. Recent Developments and Clinical Applications. J. Mater. Chem. B.

[14] Wheelton A., Mace J.S., Khan W., Anand S. (2016). Biomaterials and Fabrication to Optimise Scaffold Properties for Musculoskeletal Tissue Engineering. Curr. Stem Cell Res. Ther..

[15] Lim W.L., Liau L.L., Ng M.H., Chowdhury S.R., Law J.X. (2019). Current Progress in Tendon and Ligament Tissue Engineering. Tissue Eng. Regen. Med..

[16] Filippi M., Born G., Chaaban M., Scherberich A. (2020). Natural Polymeric Scaffolds in Bone Regeneration. Front. Bioeng. Biotechnol..

[17] Malafaya P.B., Silva G.A., Reis R.L. (2007). Natural-Origin Polymers as Carriers and Scaffolds for Biomolecules and Cell Delivery in Tissue Engineering Applications. Adv. Drug Deliv. Rev..

[18] Del Bakhshayesh A.R., Asadi N., Alihemmati A., Tayefi Nasrabadi H., Montaseri A., Davaran S., Saghati S., Akbarzadeh A., Abedelahi A. (2019). An Overview of Advanced Biocompatible and Biomimetic Materials for Creation of Replacement Structures in the Musculoskeletal Systems: Focusing on Cartilage Tissue Engineering. J. Biol. Eng..

[19] Roberts J.J., Martens P.J. (2016). Engineering Biosynthetic Cell Encapsulation Systems. Biosynthetic Polymers for Medical Applications.

[20] Spicer C.D. (2020). Hydrogel Scaffolds for Tissue Engineering: The Importance of Polymer Choice. Polym. Chem..

[21] Bao W., Li M., Yang Y., Wan Y., Wang X., Bi N., Li C. (2020). Advancements and Frontiers in the High Performance of Natural Hydrogels for Cartilage Tissue Engineering. Front. Chem..

[22] Bhattarai D.P., Aguilar L.E., Park C.H., Kim C.S. (2018). A Review on Properties of Natural and Synthetic Based Electrospun Fibrous Materials for Bone Tissue Engineering. Membranes.

[23] Tan G.Z., Zhou Y. (2020). Electrospinning of Biomimetic Fibrous Scaffolds for Tissue Engineering: A Review. Int. J. Polym. Mater. Polym. Biomater..

[24] Robb K.P., Shridhar A., Flynn L.E. (2018). Decellularized Matrices as Cell-Instructive Scaffolds to Guide Tissue-Specific Regeneration. ACS Biomater. Sci. Eng..

[25] Bose S., Koski C., Vu A.A. (2020). Additive Manufacturing of Natural Biopolymers and Composites for Bone Tissue Engineering. Mater. Horiz..

[26] Dhandayuthapani B., Yoshida Y., Maekawa T., Kumar D.S. (2011). Polymeric Scaffolds in Tissue Engineering Application: A Review. Int. J. Polym. Sci..

[27] Lavanya K., Chandran S.V., Balagangadharan K., Selvamurugan N. (2020). Temperature- and PH-Responsive Chitosan-Based Injectable Hydrogels for Bone Tissue Engineering. Mater. Sci. Eng. C.

[28] Islam S., Bhuiyan M.A.R., Islam M.N. (2017). Chitin and Chitosan: Structure, Properties and Applications in Biomedical Engineering. J. Polym. Environ..

[29] Ahmed S., Annu, Ali A., Sheikh J. (2018). A Review on Chitosan Centred Scaffolds and Their Applications in Tissue Engineering. Int. J. Biol. Macromol..

[30] Hernández-González A.C., Téllez-Jurado L., Rodríguez-Lorenzo L.M. (2020). Alginate Hydrogels for Bone Tissue Engineering, from Injectables to Bioprinting: A Review. Carbohydr. Polym..

[31] Venkatesan J., Bhatnagar I., Manivasagan P., Kang K.-H., Kim S.-K. (2015). Alginate Composites for Bone Tissue Engineering: A Review. Int. J. Biol. Macromol..

[32] Rajesh R., Dominic Ravichandran Y., Kuo Y.C., Venkatesan J., Anil S., Kim S.-K. (2017). Alginate in Bone Tissue Engineering. Seaweed Polysaccharides: Isolation, Biological and Biomedical Applications.

[33] Farokhi M., Shariatzadeh F.J., Solouk A., Mirzadeh H. (2020). Alginate Based Scaffolds for Cartilage Tissue Engineering: A Review. Int. J. Polym. Mater. Polym. Biomater..

[34] Roslan M.R., Nasir N.F.F.M., Cheng E.M., Amin N.A.M. Tissue Engineering Scaffold Based on Starch: A Review. Proceedings of the International Conference on Electrical, Electronics, and Optimization Techniques, ICEEOT 2016, Chennai, India, 3–5 March 2016.

[35] Robyt J.F. (2008). Starch: Structure, Properties, Chemistry, and Enzymology. Glycoscience.

[36] Hemamalini T., Giri Dev V.R. (2018). Comprehensive Review on Electrospinning of Starch Polymer for Biomedical Applications. Int. J. Biol. Macromol..

[37] Fakhari A., Berkland C. (2013). Applications and Emerging Trends of Hyaluronic Acid in Tissue Engineering, as a Dermal Filler and in Osteoarthritis Treatment. Acta Biomater..

[38] Hemshekhar M., Thushara R.M., Chandranayaka S., Sherman L.S., Kemparaju K., Girish K.S. (2016). Emerging Roles of Hyaluronic Acid Bioscaffolds in Tissue Engineering and Regenerative Medicine. Int. J. Biol. Macromol..

[39] Chircov C., Grumezescu A.M., Bejenaru L.E. (2018). Hyaluronic Acid-Based Scaffolds for Tissue Engineering. Rom. J. Morphol. Embryol..

[40] Collins M.N., Birkinshaw C. (2013). Hyaluronic Acid Based Scaffolds for Tissue Engineering—A Review. Carbohydr. Polym..

[41] Yang J., Shen M., Wen H., Luo Y., Huang R., Rong L., Xie J. (2020). Recent Advance in Delivery System and Tissue Engineering Applications of Chondroitin Sulfate. Carbohydr. Polym..

[42] Zarrintaj P., Manouchehri S., Ahmadi Z., Saeb M.R., Urbanska A.M., Kaplan D.L., Mozafari M. (2018). Agarose-Based Biomaterials for Tissue Engineering. Carbohydr. Polym..

[43] Khang G., Lee S.J., Kim M.S., Lee H.B. (2006). Biomaterials: Tissue Engineering and Scaffolds. Encyclopedia of Medical Devices and Instrumentation.

[44] Eslahi N., Mahmoodi A., Mahmoudi N., Zandi N., Simchi A. (2020). Processing and Properties of Nanofibrous Bacterial Cellulose-Containing Polymer Composites: A Review of Recent Advances for Biomedical Applications. Polym. Rev..

[45] Torgbo S., Sukyai P. (2018). Bacterial Cellulose-Based Scaffold Materials for Bone Tissue Engineering. Appl. Mater. Today.

[46] Halib N., Ahmad I., Grassi M., Grassi G. (2019). The Remarkable Three-Dimensional Network Structure of Bacterial Cellulose for Tissue Engineering Applications. Int. J. Pharm..

[47] Pang M., Huang Y., Meng F., Zhuang Y., Liu H., Du M., Ma Q., Wang Q., Chen Z., Chen L. (2020). Application of Bacterial Cellulose in Skin and Bone Tissue Engineering. Eur. Polym. J..

[48] Hickey R.J., Pelling A.E. (2019). Cellulose Biomaterials for Tissue Engineering. Front. Bioeng. Biotechnol..

[49] Sun G., Mao J.J. (2012). Engineering Dextran-Based Scaffolds for Drug Delivery and Tissue Repair. Nanomedicine.

[50] Maia J., Evangelista M., Gil H., Ferreira L. (2014). Dextran-Based Materials for Biomedical Applications. Carbohydrates Applications in Medicine.

[51] Varshosaz J. (2012). Dextran Conjugates in Drug Delivery. Expert Opin. Drug Deliv..

[52] Zia K.M., Tabasum S., Nasif M., Sultan N., Aslam N., Noreen A., Zuber M. (2017). A Review on Synthesis, Properties and Applications of Natural Polymer Based Carrageenan Blends and Composites. Int. J. Biol. Macromol..

[53] Zia K.M., Tabasum S., Khan M.F., Akram N., Akhter N., Noreen A., Zuber M. (2018). Recent Trends on Gellan Gum Blends with Natural and Synthetic Polymers: A Review. Int. J. Biol. Macromol..

[54] Stevens L.R., Gilmore K.J., Wallace G.G., In het Panhuis M. (2016). Tissue Engineering with Gellan Gum. Biomater. Sci..

[55] Mohammadinejad R., Kumar A., Ranjbar-Mohammadi M., Ashrafizadeh M., Han S.S., Khang G., Roveimiab Z. (2020). Recent Advances in Natural Gum-Based Biomaterials for Tissue Engineering and Regenerative Medicine: A Review. Polymers.

[56] Kumar A., Rao K.M., Han S.S. (2018). Application of Xanthan Gum as Polysaccharide in Tissue Engineering: A Review. Carbohydr. Polym..

[57] del Rodriguez-Torres M.P., Acosta-Torres L.S., Diaz-Torres L.A. (2018). Heparin-Based Nanoparticles: An Overview of Their Applications. J. Nanomater..

[58] Liang Y., Kiick K.L. (2014). Heparin-Functionalized Polymeric Biomaterials in Tissue Engineering and Drug Delivery Applications. Acta Biomater..

[59] Dong C., Lv Y. (2016). Application of Collagen Scaffold in Tissue Engineering: Recent Advances and New Perspectives. Polymers.

[60] Parenteau-Bareil R., Gauvin R., Berthod F. (2010). Collagen-Based Biomaterials for Tissue Engineering Applications. Materials.

[61] Marques C.F., Diogo G.S., Pina S., Oliveira J.M., Silva T.H., Reis R.L. (2019). Collagen-Based Bioinks for Hard Tissue Engineering Applications: A Comprehensive Review. J. Mater. Sci. Mater. Med..

[62] Silver F.H., Jaffe M., Shah R.G., Bunsell A.R. (2018). Structure and Behavior of Collagen Fibers. Handbook of Properties of Textile and Technical Fibres.

[63] Afewerki S., Sheikhi A., Kannan S., Ahadian S., Khademhosseini A. (2018). Gelatin-Polysaccharide Composite Scaffolds for 3D Cell Culture and Tissue Engineering: Towards Natural Therapeutics. Bioeng. Transl. Med..

[64] Hoque M., Nuge T., Yeow T., Nordin N., Prasad R. (2015). Gelatin Based Scaffolds for Tissue Engineering—A Review. Polym. Res. J..

[65] Kuttappan S., Mathew D., Nair M.B. (2016). Biomimetic Composite Scaffolds Containing Bioceramics and Collagen/Gelatin for Bone Tissue Engineering—A Mini Review. Int. J. Biol. Macromol..

[66] Tungkavet T., Pattavarakorn D., Sirivat A. (2012). Bio-Compatible Gelatins (Ala-Gly-Pro-Arg-Gly-Glu-4Hyp-Gly-Pro-) and Electromechanical Properties: Effects of Temperature and Electric Field. J. Polym. Res..

[67] Melke J., Midha S., Ghosh S., Ito K., Hofmann S. (2016). Silk Fibroin as Biomaterial for Bone Tissue Engineering. Acta Biomater..

[68] Koh L.-D., Cheng Y., Teng C.-P., Khin Y.-W., Loh X.-J., Tee S.-Y., Low M., Ye E., Yu H.-D., Zhang Y.-W. (2015). Structures, Mechanical Properties and Applications of Silk Fibroin Materials. Prog. Polym. Sci..

[69] Wang Y., Kim H.-J., Vunjak-Novakovic G., Kaplan D.L. (2006). Stem Cell-Based Tissue Engineering with Silk Biomaterials. Biomaterials.

[70] Nguyen T.P., Nguyen Q.V., Nguyen V.-H., Le T.-H., Huynh V.Q.N., Vo D.-V.N., Trinh Q.T., Kim S.Y., Le Q. (2019). Van Silk Fibroin-Based Biomaterials for Biomedical Applications: A Review. Polymers.

[71] Kasoju N., Bora U. (2012). Silk Fibroin in Tissue Engineering. Adv. Healthc. Mater..

[72] Shavandi A., Silva T.H., Bekhit A.A., Bekhit A.E.D.A. (2017). Keratin: Dissolution, Extraction and Biomedical Application. Biomater. Sci..

[73] Lu T.-Y., Huang W.-C., Chen Y., Baskaran N., Yu J., Wei Y. (2020). Effect of Varied Hair Protein Fractions on the Gel Properties of Keratin/Chitosan Hydrogels for the Use in Tissue Engineering. Colloids Surf. B Biointerfaces.

[74] Costa F., Silva R., Boccaccini A.R., Barbosa M.A., Cristina M., Martins L. (2018). Fibrous Protein-Based Biomaterials (Silk, Keratin, Elastin, and Resilin Proteins) for Tissue Regeneration and Repair. Peptides and Proteins as Biomaterials for Tissue Regeneration and Repair.

[75] Rouse J.G., Van Dyke M.E. (2010). A Review of Keratin-Based Biomaterials for Biomedical Applications. Materials.

[76] Chiti M.C., Dolmans M.M., Donnez J., Amorim C.A. (2017). Fibrin in Reproductive Tissue Engineering: A Review on Its Application as a Biomaterial for Fertility Preservation. Ann. Biomed. Eng..

[77] Bujoli B., Scimeca J.-C., Verron E. (2019). Fibrin as a Multipurpose Physiological Platform for Bone Tissue Engineering and Targeted Delivery of Bioactive Compounds. Pharmaceutics.

[78] Li Y., Meng H., Liu Y., Lee B.P. (2015). Fibrin Gel as an Injectable Biodegradable Scaffold and Cell Carrier for Tissue Engineering. Sci. World J..

[79] de la Puente P., Ludeña D. (2014). Cell Culture in Autologous Fibrin Scaffolds for Applications in Tissue Engineering. Exp. Cell Res..

[80] Yeo G.C., Mithieux S.M., Weiss A.S. (2018). The Elastin Matrix in Tissue Engineering and Regeneration. Curr. Opin. Biomed. Eng..

[81] Daamen W.F., Veerkamp J.H., van Hest J.C.M., van Kuppevelt T.H. (2007). Elastin as a Biomaterial for Tissue Engineering. Biomaterials.

[82] Karpiński R., Jaworski Ł., Czubacka P. (2017). The Structural and Mechanical Properties of the Bone. J. Technol. Exploit. Mech. Eng..

[83] Miller M.D., Thompson S.R. (2016). Miller’s Review of Orthopaedics.

[84] Khurana J.S. (2009). Bone Pathology.

[85] Zhang D., Wu X., Chen J., Lin K. (2018). The Development of Collagen Based Composite Scaffolds for Bone Regeneration. Bioact. Mater..

[86] Ishimoto T., Sato B., Lee J.W., Nakano T. (2017). Co-Deteriorations of Anisotropic Extracellular Matrix Arrangement and Intrinsic Mechanical Property in c-Src Deficient Osteopetrotic Mouse Femur. Bone.

[87] Viswanath B., Raghavan R., Ramamurty U., Ravishankar N. (2007). Mechanical Properties and Anisotropy in Hydroxyapatite Single Crystals. Scr. Mater..

[88] Tanaka Y., Kubota A., Matsusaki M., Duncan T., Hatakeyama Y., Fukuyama K., Quantock A.J., Yamato M., Akashi M., Nishida K. (2011). Anisotropic Mechanical Properties of Collagen Hydrogels Induced by Uniaxial-Flow for Ocular Applications. J. Biomater.Sci. Polym. Ed..

[89] Fan J., Jahed V., Klavins K. (2021). Metabolomics in Bone Research. Metabolites.

[90] Qu H., Fu H., Han Z., Sun Y. (2019). Biomaterials for Bone Tissue Engineering Scaffolds: A Review. RSC Adv..

[91] (2004). U.S. Department of HHS Bone Health and Osteoporosis: A Report of the Surgeon General. US Health Human Service.

[92] Monterde-Cruz L., Ramírez-Salazar E.G., Rico-Martínez G., Linares-González L.M., Guzmán-González R., Delgado-Cedillo E., Estrada-Villaseñor E., Valdés-Flores M., Velázquez-Cruz R., Hidalgo-Bravo A. (2020). MicroRNA Expression in Relation with Clinical Evolution of Osteosarcoma. Pathol. Res. Pract..

[93] Lu Y., Li M., Long Z., Yang D., Guo S., Li J., Liu D., Gao P., Chen G., Lu X. (2019). Collagen/β-TCP Composite as a Bone-Graft Substitute for Posterior Spinal Fusion in Rabbit Model: A Comparison Study. Biomed. Mater..

[94] Zheng H., Bai Y., Shih M.S., Hoffmann C., Peters F., Waldner C., Hübner W.D. (2014). Effect of a β-TCP Collagen Composite Bone Substitute on Healing of Drilled Bone Voids in the Distal Femoral Condyle of Rabbits. J. Biomed. Mater. Res. Part B Appl. Biomater..

[95] Guerrero-Gironés J., Alcaina-Lorente A., Ortiz-Ruiz C., Ortiz-Ruiz E., Pecci-Lloret M.P., Ortiz-Ruiz A.J., Rodríguez-Lozano F.J., Pecci-Lloret M.R. (2021). Biocompatibility of a Ha/Β-tcp/c Scaffoldas a Pulp-capping Agent for Vital Pulp Treatment: An in Vivo Study in Rat Molars. Int. J. Environ. Res. Public Health.

[96] Ebrahimi M., Botelho M.G., Dorozhkin S.V. (2017). Biphasic Calcium Phosphates Bioceramics (HA/TCP): Concept, Physicochemical Properties and the Impact of Standardization of Study Protocols in Biomaterials Research. Mater. Sci. Eng. C.

[97] Mohamed N., Rahamana, Daya D.E., Balb B.S., Fuc Q., Junga S.B., Lynda F., Bonewalde A.P.T. (2011). Bioactive Glass in Tissue Engineering. Acta Biomater..

[98] Gerhardt L.C., Boccaccini A.R. (2010). Bioactive Glass and Glass-Ceramic Scaffolds for Bone Tissue Engineering. Materials.

[99] Ferreira S.A., Young G., Jones J.R., Rankin S. (2021). Bioglass/Carbonate Apatite/Collagen Composite Scaffold Dissolution Products Promote Human Osteoblast Differentiation. Mater. Sci. Eng. C.

[100] Hamzah M.S.A., Ng C., Zulkarnain N.I.S., Majid H.A., Razak S.I.A., Nayan N.H.M. (2021). Entrapment of Collagen on Polylactic Acid 3D Scaffold Surface as a Potential Artificial Bone Replacement. Mater. Today Proc..

[101] Dewey M.J., Nosatov A.V., Subedi K., Shah R., Jakus A., Harley B.A.C. (2021). Inclusion of a 3D-Printed Hyperelastic Bone Mesh Improves Mechanical and Osteogenic Performance of a Mineralized Collagen Scaffold. Acta Biomater..

[102] Oh G.W., Nguyen V.T., Heo S.Y., Ko S.C., Kim C.S., Park W.S., Choi I.W., Jung W.K. (2021). 3D PCL/Fish Collagen Composite Scaffolds Incorporating Osteogenic Abalone Protein Hydrolysates for Bone Regeneration Application: In Vitro and in Vivo Studies. J. Biomater. Sci. Polym. Ed..

[103] Soufdoost R.S., Yazdanian M., Tahmasebi E., Yazdanian A., Tebyanian H., Karami A., Nourani M.R., Panahi Y. (2019). In Vitro and in Vivo Evaluation of Novel Tadalafil/β-TCP/Collagen Scaffold for Bone Regeneration: A Rabbit Critical-Size Calvarial Defect Study. Biocybern. Biomed. Eng..

[104] Maier J. (2004). High Concentrations of Magnesium Modulate Vascular Endothelial Cell Behaviour In Vitro. Biochim. Et Biophys. Acta BBA Mol. Basis Dis..

[105] Lin K., Zhou Y., Zhou Y., Qu H., Chen F., Zhu Y., Chang J. (2011). Biomimetic Hydroxyapatite Porous Microspheres with Co-Substituted Essential Trace Elements: Surfactant-Free Hydrothermal Synthesis, Enhanced Degradation and Drug Release. J. Mater. Chem..

[106] Minardi S., Taraballi F., Cabrera F.J., Van Eps J., Wang X., Gazze S.A., Fernandez-Mourev J.S., Tampieri A., Francis L., Weiner B.K. (2019). Biomimetic Hydroxyapatite/Collagen Composite Drives Bone Niche Recapitulation in a Rabbit Orthotopic Model. Mater. Today Bio.

[107] Antoniac I.V., Antoniac A., Vasile E., Tecu C., Fosca M., Yankova V.G., Rau J.V. (2021). In Vitro Characterization of Novel Nanostructured Collagen-Hydroxyapatite Composite Scaffolds Doped with Magnesium with Improved Biodegradation Rate for Hard Tissue Regeneration. Bioact. Mater..

[108] Ryan E.J., Ryan A.J., González-Vázquez A., Philippart A., Ciraldo F.E., Hobbs C., Nicolosi V., Boccaccini A.R., Kearney C.J., O’Brien F.J. (2019). Collagen Scaffolds Functionalised with Copper-Eluting Bioactive Glass Reduce Infection and Enhance Osteogenesis and Angiogenesis Both In Vitro and In Vivo. Biomaterials.

[109] Jing Z., Wu Y., Su W., Tian M., Jiang W., Cao L., Zhao L., Zhao Z. (2017). Carbon Nanotube Reinforced Collagen/Hydroxyapatite Scaffolds Improve Bone Tissue Formation In Vitro and In Vivo. Ann. Biomed. Eng..

[110] Ju T., Zhao Z., Ma L., Li W., Li S., Zhang J. (2021). Cyclic Adenosine Monophosphate-Enhanced Calvarial Regeneration by Bone Marrow-Derived Mesenchymal Stem Cells on a Hydroxyapatite/Gelatin Scaffold. ACS Omega.

[111] Rezaei H., Shahrezaee M., Jalali Monfared M., Ghorbani F., Zamanian A., Sahebalzamani M. (2021). Mussel-Inspired Polydopamine Induced the Osteoinductivity to Ice-Templating PLGA–Gelatin Matrix for Bone Tissue Engineering Application. Biotechnol. Appl. Biochem..

[112] Jahangir S., Hosseini S., Mostafaei F., Sayahpour F.A., Eslaminejad M.B. (2019). 3D-Porous β-Tricalcium Phosphate–Alginate–Gelatin Scaffold with DMOG Delivery Promotes Angiogenesis and Bone Formation in Rat Calvarial Defects. J. Mater. Sci. Mater. Med..

[113] Gautam S., Sharma C., Purohit S.D., Singh H., Dinda A.K., Potdar P.D., Chou C.F., Mishra N.C. (2021). Gelatin-Polycaprolactone-Nanohydroxyapatite Electrospun Nanocomposite Scaffold for Bone Tissue Engineering. Mater. Sci. Eng. C.

[114] Lu H., Pan X., Hu M., Zhang J., Yu Y., Hu X., Jiang K. (2021). Fabrication of Graphene/Gelatin/Chitosan/Tricalcium Phosphate 3D Printed Scaffolds for Bone Tissue Regeneration Applications. Appl. Nanosci..

[115] Qu L., Dubey N., Ribeiro J.S., Bordini E.A.F., Ferreira J.A., Xu J., Castilho R.M., Bottino M.C. (2021). Metformin-Loaded Nanospheres-Laden Photocrosslinkable Gelatin Hydrogel for Bone Tissue Engineering. J. Mech. Behav. Biomed. Mater..

[116] Wang Y., Cao X., Ma M., Lu W., Zhang B., Guo Y. (2020). A GelMA-PEGDA-NHA Composite Hydrogel for Bone Tissue Engineering. Materials.

[117] Ramírez Rodríguez G., Patrício T., Delgado López J. (2019). Natural Polymers for Bone Repair. Bone Repair Biomaterials.

[118] Aguilar A., Zein N., Harmouch E., Hafdi B., Bornert F., Damien O., Clauss F., Fioretti F., Huck O., Benkirane-jessel N. (2019). Application of Chitosan in Bone and Dental Engineering. Biodental Eng. V.

[119] Brun P., Zamuner A., Battocchio C., Cassari L., Todesco M., Graziani V., Iucci G., Marsotto M., Tortora L., Secchi V. (2021). Bio-Functionalized Chitosan for Bone Tissue Engineering. Int. J. Mol. Sci..

[120] Balagangadharan K., Trivedi R., Vairamani M., Selvamurugan N. (2019). Sinapic Acid-Loaded Chitosan Nanoparticles in Polycaprolactone Electrospun Fibers for Bone Regeneration in Vitro and in Vivo. Carbohydr. Polym..

[121] Radwan N.H., Nasr M., Ishak R.A.H., Abdeltawab N.F., Awad G.A.S. (2020). Chitosan-Calcium Phosphate Composite Scaffolds for Control of Post-Operative Osteomyelitis: Fabrication, Characterization, and In Vitro–In Vivo Evaluation. Carbohydr. Polym..

[122] Bari E., Scocozza F., Perteghella S., Sorlini M., Auricchio F., Torre M.L., Conti M. (2021). 3d Bioprinted Scaffolds Containing Mesenchymal Stem/Stromal Lyosecretome: Next Generation Controlled Release Device for Bone Regenerative Medicine. Pharmaceutics.

[123] Zhang J., Eyisoylu H., Qin X.H., Rubert M., Müller R. (2021). 3D Bioprinting of Graphene Oxide-Incorporated Cell-Laden Bone Mimicking Scaffolds for Promoting Scaffold Fidelity, Osteogenic Differentiation and Mineralization. Acta Biomater..

[124] Keshavarz M., Alizadeh P. (2021). On the Role of Alginate Coating on the Mechanical and Biological Properties of 58S Bioactive Glass Scaffolds. Int. J. Biol. Macromol..

[125] Zhou W., Li Q., Ma R., Huang W., Zhang X., Liu Y., Xu Z., Zhang L., Li M., Zhu C. (2021). Modified Alginate-Based Hydrogel as a Carrier of the CB2 Agonist JWH133 for Bone Engineering. ACS Omega.

[126] Zhang H., Cai Q., Zhu Y., Zhu W. (2021). A Simple Hydrogel Scaffold with Injectability, Adhesivity and Osteogenic Activity for Bone Regeneration. Biomater. Sci..

[127] Garske D.S., Schmidt-Bleek K., Ellinghaus A., Dienelt A., Gu L., Mooney D.J., Duda G.N., Cipitria A. (2020). Alginate Hydrogels for In Vivo Bone Regeneration: The Immune Competence of the Animal Model Matters. Tissue Eng. Part A.

[128] Erickson C.B., Newsom J.P., Fletcher N.A., Feuer Z.M., Yu Y., Rodriguez-Fontan F., Hadley Miller N., Krebs M.D., Payne K.A. (2020). In Vivo Degradation Rate of Alginate–Chitosan Hydrogels Influences Tissue Repair Following Physeal Injury. J. Biomed. Mater. Res. Part B Appl. Biomater..

[129] Ke X., Dong Z., Tang S., Chu W., Zheng X., Zhen L., Chen X., Ding C., Luo J., Li J. (2020). A Natural Polymer Based Bioadhesive with Self-Healing Behavior and Improved Antibacterial Properties. Biomater. Sci..

[130] Yang N., Moore M.J., Michael P.L., Santos M., Lam Y.T., Bao S., Ng M.K.C., Rnjak-Kovacina J., Tan R.P., Wise S.G. (2021). Silk Fibroin Scaffold Architecture Regulates Inflammatory Responses and Engraftment of Bone Marrow-Mononuclear Cells. Adv. Healthc. Mater..

[131] Teramoto H., Shirakawa M., Tamada Y. (2020). Click Decoration of Bombyx Mori Silk Fibroin for Cell Adhesion Control. Molecules.

[132] Akrami-Hasan-Kohal M., Eskandari M., Solouk A. (2021). Silk Fibroin Hydrogel/Dexamethasone Sodium Phosphate Loaded Chitosan Nanoparticles as a Potential Drug Delivery System. Colloids Surf. B Biointerfaces.

[133] Zheng H., Zuo B. (2021). Functional Silk Fibroin Hydrogels: Preparation, Properties and Applications. J. Mater. Chem. B.

[134] Wu M., Han Z., Liu W., Yao J., Zhao B., Shao Z., Chen X. (2021). Silk-Based Hybrid Microfibrous Mats as Guided Bone Regeneration Membranes. J. Mater. Chem. B.

[135] Zheng X., Ke X., Yu P., Wang D., Pan S., Yang J., Ding C., Xiao S., Luo J., Li J. (2020). A Facile Strategy to Construct Silk Fibroin Based GTR Membranes with Appropriate Mechanical Performance and Enhanced Osteogenic Capacity. J. Mater. Chem. B.

[136] McNamara S.L., McCarthy E.M., Schmidt D.F., Johnston S.P., Kaplan D.L. (2021). Rheological Characterization, Compression, and Injection Molding of Hydroxyapatite-Silk Fibroin Composites. Biomaterials.

[137] Saleem M., Rasheed S., Yougen C. (2020). Silk Fibroin/Hydroxyapatite Scaffold: A Highly Compatible Material for Bone Regeneration. Sci. Technol. Adv. Mater..

[138] Du X., Wei D., Huang L., Zhu M., Zhang Y., Zhu Y. (2019). 3D Printing of Mesoporous Bioactive Glass/Silk Fibroin Composite Scaffolds for Bone Tissue Engineering. Mater. Sci. Eng. C.

[139] Xiao L., Wu M., Yan F., Xie Y., Liu Z., Huang H., Yang Z., Yao S., Cai L. (2021). A Radial 3D Polycaprolactone Nanofiber Scaffold Modified by Biomineralization and Silk Fibroin Coating Promote Bone Regeneration In Vivo. Int. J. Biol. Macromol..

[140] Chen P., Liu L., Pan J., Mei J., Li C., Zheng Y. (2019). Biomimetic Composite Scaffold of Hydroxyapatite/Gelatin-Chitosan Core-Shell Nanofibers for Bone Tissue Engineering. Mater. Sci. Eng. C.

[141] Purohit S.D., Bhaskar R., Singh H., Yadav I., Gupta M.K., Mishra N.C. (2019). Development of a Nanocomposite Scaffold of Gelatin–Alginate–Graphene Oxide for Bone Tissue Engineering. Int. J. Biol. Macromol..

[142] Thomas A., Bera J. (2019). Preparation and Characterization of Gelatin-Bioactive Glass Ceramic Scaffolds for Bone Tissue Engineering. J. Biomater. Sci. Polym. Ed..

[143] Sharifi F., Atyabi S.M., Norouzian D., Zandi M., Irani S., Bakhshi H. (2018). Polycaprolactone/Carboxymethyl Chitosan Nanofibrous Scaffolds for Bone Tissue Engineering Application. Int. J. Biol. Macromol..

[144] Dai C., Li Y., Pan W., Wang G., Huang R., Bu Y., Liao X., Guo K., Gao F. (2020). Three-Dimensional High-Porosity Chitosan/Honeycomb Porous Carbon/Hydroxyapatite Scaffold with Enhanced Osteoinductivity for Bone Regeneration. ACS Biomater. Sci. Eng..

[145] Shi D., Shen J., Zhang Z., Shi C., Chen M., Gu Y., Liu Y. (2019). Preparation and Properties of Dopamine-Modified Alginate/Chitosan–Hydroxyapatite Scaffolds with Gradient Structure for Bone Tissue Engineering. J. Biomed. Mater. Res. Part A.

[146] Saini R.K., Bagri L.P., Bajpai A.K. (2019). Nano-Silver Hydroxyapatite Based Antibacterial 3D Scaffolds of Gelatin/Alginate/Poly (Vinyl Alcohol) for Bone Tissue Engineering Applications. Colloids Surf. B Biointerfaces.

[147] Echave M.C., Pimenta-Lopes C., Pedraz J.L., Mehrali M., Dolatshahi-Pirouz A., Ventura F., Orive G. (2019). Enzymatic Crosslinked Gelatin 3D Scaffolds for Bone Tissue Engineering. Int. J. Pharm..

[148] Rajzer I., Kurowska A., Jabłoński A., Jatteau S., Śliwka M., Ziąbka M., Menaszek E. (2018). Layered Gelatin/PLLA Scaffolds Fabricated by Electrospinning and 3D Printing- for Nasal Cartilages and Subchondral Bone Reconstruction. Mater. Des..

[149] Panzavolta S., Torricelli P., Casolari S., Parrilli A., Fini M., Bigi A. (2018). Strontium-Substituted Hydroxyapatite-Gelatin Biomimetic Scaffolds Modulate Bone Cell Response. Macromol. Biosci..

[150] Sattary M., Rafienia M., Kazemi M., Salehi H., Mahmoudzadeh M. (2019). Promoting Effect of Nano Hydroxyapatite and Vitamin D3 on the Osteogenic Differentiation of Human Adipose-Derived Stem Cells in Polycaprolactone/Gelatin Scaffold for Bone Tissue Engineering. Mater. Sci. Eng. C.

[151] Sun J.L., Jiao K., Niu L.-N., Jiao Y., Song Q., Shen L.-J., Tay F.R., Chen J.-H. (2017). 5Intrafibrillar Silicified Collagen Scaffold Modulates Monocyte to Promote Cell Homing, Angiogenesis and Bone Regeneration. Biomaterials.

[152] Wang Z., Lin M., Xie Q., Sun H., Huang Y., Zhang D.D., Yu Z., Bi X., Chen J., Wang J. (2016). Electrospun Silk Fibroin/Poly(Lactide-Co-ε-Caprolactone) Nanofibrous Scaffolds for Bone Regeneration. Int. J. Nanomed..

[153] Yao J., Wang Y., Ma W., Dong W., Zhang M., Sun D. (2019). Dual-Drug-Loaded Silk Fibroin/PLGA Scaffolds for Potential Bone Regeneration Applications. J. Nanomater..

[154] He J.X., Tan W.L., Han Q.M., Cui S.Z., Shao W., Sang F. (2016). Fabrication of Silk Fibroin/Cellulose Whiskers–Chitosan Composite Porous Scaffolds by Layer-by-Layer Assembly for Application in Bone Tissue Engineering. J. Mater. Sci..

[155] Luetchford K.A., Chaudhuri J.B., De Bank P.A. (2020). Silk Fibroin/Gelatin Microcarriers as Scaffolds for Bone Tissue Engineering. Mater. Sci. Eng. C.

[156] Benedini L., Laiuppa J., Santillán G., Baldini M., Messina P. (2020). Antibacterial Alginate/Nano-Hydroxyapatite Composites for Bone Tissue Engineering: Assessment of Their Bioactivity, Biocompatibility, and Antibacterial Activity. Mater. Sci. Eng. C.

[157] Zheng A., Cao L., Liu Y., Wu J., Zeng D., Hu L., Zhang X., Jiang X. (2018). Biocompatible Silk/Calcium Silicate/Sodium Alginate Composite Scaffolds for Bone Tissue Engineering. Carbohydr. Polym..

[158] Wang P., Song Y., Weir M.D., Sun J., Zhao L., Simon C.G., Xu H.H.K. (2016). A Self-Setting IPSMSC-Alginate-Calcium Phosphate Paste for Bone Tissue Engineering. Dent. Mater..

[159] Luo Y., Li Y., Qin X., Wa Q. (2018). 3D Printing of Concentrated Alginate/Gelatin Scaffolds with Homogeneous Nano Apatite Coating for Bone Tissue Engineering. Mater. Des..

[160] Scott W., Stevens J., Binder-Macleod S.A. (2001). Human Skeletal Muscle Fiber Type Classifications. Phys. Ther..

[161] Relaix F., Bencze M., Borok M.J., Der Vartanian A., Gattazzo F., Mademtzoglou D., Perez-Diaz S., Prola A., Reyes-Fernandez P.C., Rotini A. (2021). Perspectives on Skeletal Muscle Stem Cells. Nat. Commun..

[162] Ostrovidov S., Salehi S., Costantini M., Suthiwanich K., Ebrahimi M., Sadeghian R.B., Fujie T., Shi X., Cannata S., Gargioli C. (2019). 3D Bioprinting in Skeletal Muscle Tissue Engineering. Small.

[163] Fischer K.M., Scott T.E., Browe D.P., McGaughey T.A., Wood C., Wolyniak M.J., Freeman J.W. (2020). Hydrogels for Skeletal Muscle Regeneration. Regen. Eng. Transl. Med..

[164] Liu J., Saul D., Böker K.O., Ernst J., Lehman W., Schilling A.F. (2018). Current Methods for Skeletal Muscle Tissue Repair and Regeneration. BioMed Res. Int..

[165] Gholobova D., Terrie L., Gerard M., Declercq H., Thorrez L. (2020). Vascularization of Tissue-Engineered Skeletal Muscle Constructs. Biomaterials.

[166] Zhuang P., An J., Chua C.K., Tan L.P. (2020). Bioprinting of 3D in Vitro Skeletal Muscle Models: A Review. Mater. Des..

[167] del Carmen Ortuño-Costela M., García-López M., Cerrada V., Gallardo M.E. (2019). IPSCs: A Powerful Tool for Skeletal Muscle Tissue Engineering. J. Cell. Mol. Med..

[168] Gillies A.R., Lieber R.L. (2011). Structure and Function of the Skeletal Muscle Extracellular Matrix. Muscle Nerve.

[169] Csapo R., Gumpenberger M., Wessner B. (2020). Skeletal Muscle Extracellular Matrix—What Do We Know About Its Composition, Regulation, and Physiological Roles? A Narrative Review. Front. Physiol..

[170] Takala T.E., Virtanen P. (2000). Biochemical Composition of Muscle Extracellular Matrix: The Effect of Loading. Scand. J. Med. Sci. Sports.

[171] Thorsteinsdottir S., Deries M., Cachaço A.S., Bajanca F. (2011). The Extracellular Matrix Dimension of Skeletal Muscle Development. Dev. Biol..

[172] Baker H.B., Passipieri J.A., Siriwardane M., Ellenburg M.D., Vadhavkar M., Bergman C.R., Saul J.M., Tomblyn S., Burnett L., Christ G.J. (2017). Cell and Growth Factor-Loaded Keratin Hydrogels for Treatment of Volumetric Muscle Loss in a Mouse Model. Tissue Eng. Part A.

[173] Tomblyn S., Kneller E.L.P., Walker S.J., Ellenburg M.D., Kowalczewski C.J., Van Dyke M., Burnett L., Saul J.M. (2016). Keratin Hydrogel Carrier System for Simultaneous Delivery of Exogenous Growth Factors and Muscle Progenitor Cells. J. Biomed. Mater. Res. Part B Appl. Biomater..

[174] Passipieri J.A., Baker H.B., Siriwardane M., Ellenburg M.D., Vadhavkar M., Saul J.M., Tomblyn S., Burnett L., Christ G.J. (2017). Keratin Hydrogel Enhances In Vivo Skeletal Muscle Function in a Rat Model of Volumetric Muscle Loss. Tissue Eng. Part A.

[175] Wan X., Li P., Jin X., Su F., Shen J., Yuan J. (2020). Poly(ε-Caprolactone)/Keratin/Heparin/VEGF Biocomposite Mats for Vascular Tissue Engineering. J. Biomed. Mater. Res. Part A.

[176] Dou J., Wang Y., Jin X., Li P., Wang L., Yuan J., Shen J. (2020). PCL/Sulfonated Keratin Mats for Vascular Tissue Engineering Scaffold with Potential of Catalytic Nitric Oxide Generation. Mater. Sci. Eng. C.

[177] Li P., Wang Y., Jin X., Dou J., Han X., Wan X., Yuan J., Shen J. (2020). Fabrication of PCL/Keratin Composite Scaffolds for Vascular Tissue Engineering with Catalytic Generation of Nitric Oxide Potential. J. Mater. Chem. B.

[178] Korniłłowicz-Kowalska T., Bohacz J. (2011). Biodegradation of Keratin Waste: Theory and Practical Aspects. Waste Manag..

[179] Panayi A.C., Smit L., Hays N., Udeh K., Endo Y., Li B., Sakthivel D., Tamayol A., Neppl R.L., Orgill D.P. (2020). A Porous Collagen-GAG Scaffold Promotes Muscle Regeneration Following Volumetric Muscle Loss Injury. Wound Repair Regen..

[180] Wang H.D., Lough D.M., Kurlander D.E., Lopez J., Quan A., Kumar A.R. (2019). Muscle-Derived Stem Cell-Enriched Scaffolds Are Capable of Enhanced Healing of a Murine Volumetric Muscle Loss Defect. Plast. Reconstr. Surg..

[181] Quarta M., Cromie M., Chacon R., Blonigan J., Garcia V., Akimenko I., Hamer M., Paine P., Stok M., Shrager J.B. (2017). Bioengineered Constructs Combined with Exercise Enhance Stem Cell-Mediated Treatment of Volumetric Muscle Loss. Nat. Commun..

[182] Zarei M., Samimi A., Khorram M., Abdi M.M., Golestaneh S.I. (2021). Fabrication and Characterization of Conductive Polypyrrole/Chitosan/Collagen Electrospun Nanofiber Scaffold for Tissue Engineering Application. Int. J. Biol. Macromol..

[183] Hwangbo H., Kim W.J., Kim G.H. (2021). Lotus-Root-Like Microchanneled Collagen Scaffold. ACS Appl. Mater. Interfaces.

[184] Perez-Puyana V., Wieringa P., Yuste Y., de la Portilla F., Guererro A., Romero A., Moroni L. (2021). Fabrication of Hybrid Scaffolds Obtained from Combinations of PCL with Gelatin or Collagen via Electrospinning for Skeletal Muscle Tissue Engineering. J. Biomed. Mater. Res. Part A.

[185] Basurto I.M., Mora M.A., Christ G.J., Caliari S.R. Aligned and Conductive 3D Collagen Scaffolds for Skeletal Muscle Tissue Engineering. Proceedings of the Transactions of the Annual Meeting of the Society for Biomaterials and the Annual International Biomaterials Symposium.

[186] Nakayama K.H., Quarta M., Paine P., Alcazar C., Karakikes I., Garcia V., Abilez O.J., Calvo N.S., Simmons C.S., Rando T.A. (2019). Treatment of Volumetric Muscle Loss in Mice Using Nanofibrillar Scaffolds Enhances Vascular Organization and Integration. Commun. Biol..

[187] Quigley A.F., Cornock R., Mysore T., Foroughi J., Kita M., Razal J.M., Crook J., Moulton S.E., Wallace G.G., Kapsa R.M.I. (2020). Wet-Spun Trojan Horse Cell Constructs for Engineering Muscle. Front. Chem..

[188] Distler T., Solisito A.A., Schneidereit D., Friedrich O., Detsch R., Boccaccini A.R. (2020). 3D Printed Oxidized Alginate-Gelatin Bioink Provides Guidance for C2C12 Muscle Precursor Cell Orientation and Differentiation via Shear Stress During Bioprinting. IOP Publ. Biofabr. J. Biofabr..

[189] Ansari S., Chen C., Xu X., Annabi N., Zadeh H.H., Wu B.M., Khademhosseini A., Shi S., Moshaverinia A. (2016). Muscle Tissue Engineering Using Gingival Mesenchymal Stem Cells Encapsulated in Alginate Hydrogels Containing Multiple Growth Factors. Ann. Biomed. Eng..

[190] Ciriza J., Rodríguez-Romano A., Nogueroles I., Gallego-Ferrer G., Cabezuelo R.M., Pedraz J.L., Rico P. (2021). Borax-Loaded Injectable Alginate Hydrogels Promote Muscle Regeneration in Vivo after an Injury. Mater. Sci. Eng. C.

[191] Pęziński M., Daszczuk P., Pradhan B.S., Lochmüller H., Prószyński T.J. (2020). An Improved Method for Culturing Myotubes on Laminins for the Robust Clustering of Postsynaptic Machinery. Sci. Rep..

[192] Marcinczyk M., Dunn A., Haas G., Madsen J., Scheidt R., Patel K., Talovic M., Garg K. (2019). The Effect of Laminin-111 Hydrogels on Muscle Regeneration in a Murine Model of Injury. Tissue Eng. Part A.

[193] Gilbert-Honick J., Ginn B., Zhang Y., Salehi S., Wagner K.R., Mao H.Q., Grayson W.L. (2018). Adipose-Derived Stem/Stromal Cells on Electrospun Fibrin Microfiber Bundles Enable Moderate Muscle Reconstruction in a Volumetric Muscle Loss Model. Cell Transplant..

[194] Russell C.S., Mostafavi A., Quint J.P., Panayi A.C., Baldino K., Williams T.J., Daubendiek J.G., Sánchez V.H., Bonick Z., Trujillo-Miranda M. (2020). In Situ Printing of Adhesive Hydrogel Scaffolds for the Treatment of Skeletal Muscle Injuries. ACS Appl. Bio Mater..

[195] Hanjun H., Hyeongjin L., Eun-Ju J., JaeYoon L., Yunju J., Dongryeol R., GeunHyung K. (2022). Bio-printing of aligned GelMa-based cell-laden structure for muscle tissue regeneration. Bioact. Mater..

[196] Basurto I.M., Mora M.A., Christ G.J., Caliari S.R. (2019). Aligned and Conductive 3D Collagen Scaffolds for Skeletal Muscle Tissue Engineering. Trans. Annu. Meet. Soc. Biomater. Annu. Int. Biomater. Symp..

[197] Aguilar-Agon K.W., Capel A.J., Martin N.R.W., Player D.J., Lewis M.P. (2019). Mechanical Loading Stimulates Hypertrophy in Tissue-Engineered Skeletal Muscle: Molecular and Phenotypic Responses. J. Cell. Physiol..

[198] Yi H., Forsythe S., He Y., Liu Q., Xiong G., Wei S., Li G., Atala A., Skardal A., Zhang Y. (2017). Tissue-Specific Extracellular Matrix Promotes Myogenic Differentiation of Human Muscle Progenitor Cells on Gelatin and Heparin Conjugated Alginate Hydrogels. Acta Biomater..

[199] Pumberger M., Qazi T.H., Ehrentraut M.C., Textor M., Kueper J., Stoltenburg-Didinger G., Winkler T., von Roth P., Reinke S., Borselli C. (2016). Synthetic Niche to Modulate Regenerative Potential of MSCs and Enhance Skeletal Muscle Regeneration.

[200] Poveda-Reyes S., Moulisova V., Sanmartín-Masiá E., Quintanilla-Sierra L., Salmerón-Sánchez M., Ferrer G.G. (2016). Gelatin—Hyaluronic Acid Hydrogels with Tuned Stiffness to Counterbalance Cellular Forces and Promote Cell Differentiation. Macromol. Biosci..

[201] Guo Y., Gilbert-Honick J., Somers S.M., Mao H.Q., Grayson W.L. (2019). Modified Cell-Electrospinning for 3D Myogenesis of C2C12s in Aligned Fibrin Microfiber Bundles. Biochem. Biophys. Res. Commun..

[202] Acosta F.M., Jia U.T.A., Stojkova K., Pacelli S., Brey E.M., Rathbone C. (2020). Divergent Effects of Myogenic Differentiation and Diabetes on the Capacity for Muscle Precursor Cell Adipogenic Differentiation in a Fibrin Matrix. Biochem. Biophys. Res. Commun..

[203] Marcinczyk M., Elmashhady H., Talovic M., Dunn A., Bugis F., Garg K. (2017). Laminin-111 Enriched Fibrin Hydrogels for Skeletal Muscle Regeneration. Biomaterials.

[204] Somers S.M., Zhang N.Y., Morrissette-McAlmon J.B.F., Tran K., Mao H.Q., Grayson W.L. (2019). Myoblast Maturity on Aligned Microfiber Bundles at the Onset of Strain Application Impacts Myogenic Outcomes. Acta Biomater..

[205] Carnes M.E., Pins G.D. (2020). Etching Anisotropic Surface Topography onto Fibrin Microthread Scaffolds for Guiding Myoblast Alignment. J. Biomed. Mater. Res. Part B Appl. Biomater..

[206] Thorrez L., DiSano K., Shansky J., Vandenburgh H. (2018). Engineering of Human Skeletal Muscle with an Autologous Deposited Extracellular Matrix. Front. Physiol..

[207] Chen X., Du W., Cai Z., Ji S., Dwivedi M., Chen J., Zhao G., Chu J. (2020). Uniaxial Stretching of Cell-Laden Microfibers for Promoting C2C12 Myoblasts Alignment and Myofibers Formation. ACS Appl. Mater. Interfaces.

[208] Hejbøl E.K., Sellathurai J., Nair P.D., Schrøder H.D. (2017). Injectable Scaffold Materials Differ in Their Cell Instructive Effects on Primary Human Myoblasts. J. Tissue Eng..

[209] Attalla R., Puersten E., Jain N., Selvaganapathy P.R. (2019). 3D Bioprinting of Heterogeneous Bi- and Tri-Layered Hollow Channels within Gel Scaffolds Using Scalable Multi-Axial Microfluidic Extrusion Nozzle. Biofabrication.

[210] Witt R., Weigand A., Boos A.M., Cai A., Dippold D., Boccaccini A.R., Schubert D.W., Hardt M., Lange C., Arkudas A. (2017). Mesenchymal Stem Cells and Myoblast Differentiation under HGF and IGF-1 Stimulation for 3D Skeletal Muscle Tissue Engineering. BMC Cell Biol..

[211] Dong Y., Li Y., Zhang C., Chen H., Liu L., Chen S. (2020). Effects of SW033291 on the Myogenesis of Muscle-Derived Stem Cells and Muscle Regeneration. Stem Cell Res. Ther..

[212] Kim W.J., Lee H., Lee J.U., Atala A., Yoo J.J., Lee S.J., Kim G.H. (2020). Efficient Myotube Formation in 3D Bioprinted Tissue Construct by Biochemical and Topographical Cues. Biomaterials.

[213] Williams N.P., Rhodehamel M., Yan C., Smith A.S.T., Jiao A., Murry C.E., Scatena M., Kim D.H. (2020). Engineering Anisotropic 3D Tubular Tissues with Flexible Thermoresponsive Nanofabricated Substrates. Biomaterials.

[214] Fischetti T., Celikkin N., Negrini N.C., Farè S., Swieszkowski W. (2020). Tripolyphosphate-Crosslinked Chitosan/Gelatin Biocomposite Ink for 3D Printing of Uniaxial Scaffolds. Front. Bioeng. Biotechnol..

[215] Seyedmahmoud R., Çelebi-Saltik B., Barros N., Nasiri R., Banton E., Shamloo A., Ashammakhi N., Dokmeci R.M., Ahadian S. (2019). Three-Dimensional Bioprinting of Functional Skeletal Muscle Tissue Using Gelatin. Micromachines.

[216] Jensen J.H., Cakal S.D., Li J., Pless C.J., Radeke C., Jepsen M.L., Jensen T.E., Dufva M., Lind J.U. (2020). Large-Scale Spontaneous Self-Organization and Maturation of Skeletal Muscle Tissues on Ultra-Compliant Gelatin Hydrogel Substrates. Sci. Rep..

[217] Simsa R., Yuen J., Stout A., Rubio N., Fogelstrand P., Kaplan D.L. (2019). Extracellular Heme Proteins Influence Bovine Myosatellite Cell Proliferation and the Color of Cell-Based Meat. Foods.

[218] Denes L.T., Riley L.A., Mijares J.R., Arboleda J.D., McKee K., Esser K.A., Wang E.T. (2019). Culturing C2C12 Myotubes on Micromolded Gelatin Hydrogels Accelerates Myotube Maturation. Skelet. Muscle.

[219] Asahara H., Inui M., Lotz M.K. (2017). Tendons and Ligaments: Connecting Developmental Biology to Musculoskeletal Disease Pathogenesis. J. Bone Miner. Res..

[220] Beldjilali-Labro M., Garcia Garcia A., Farhat F., Bedoui F., Grosset J.-F., Dufresne M., Legallais C. (2018). Biomaterials in Tendon and Skeletal Muscle Tissue Engineering: Current Trends and Challenges. Materials.

[221] Uquillas J.A., Pacelli S., Kobayashi S., Uquillas S. (2017). Musculoskeletal Tissue Engineering: Tendon, Ligament, and Skeletal Muscle Replacement and Repair. Tissue Engineering for Artificial Organs.

[222] Govoni M., Muscari C., Lovecchio J., Guarnieri C., Giordano E. (2016). Mechanical Actuation Systems for the Phenotype Commitment of Stem Cell-Based Tendon and Ligament Tissue Substitutes. Stem Cell Rev. Rep..

[223] Smith R., Carr A., Dakin S., Snelling S., Yapp C., Hakimi O. (2016). The Response of Tenocytes to Commercial Scaffolds Used for Rotator Cuff Repair. Eur. Cells Mater..

[224] Wunderli S.L., Blache U., Snedeker J.G. (2020). Tendon Explant Models for Physiologically Relevant In Vitro Study of Tissue Biology—A Perspective. Connect. Tissue Res..

[225] Liu W., Webster T.J. (2016). Toxicity and Biocompatibility Properties of Nanocomposites for Musculoskeletal Tissue Regeneration. Nanocomposites for Musculoskeletal Tissue Regeneration.

[226] Anjana J., Deepthi S., Shalumon K.T., Mony U., Chen J.P., Jayakumar R. (2018). Nanoengineered Biomaterials for Tendon/Ligament Regeneration. Nanoengineered Biomaterials for Regenerative Medicine.

[227] Sensini A., Gualandi C., Focarete M.L., Belcari J., Zucchelli A., Boyle L., Reilly G.C., Kao A.P., Tozzi G., Cristofolini L. (2019). Multiscale Hierarchical Bioresorbable Scaffolds for the Regeneration of Tendons and Ligaments. Biofabrication.

[228] Gereke T., Döbrich O., Aibibu D., Nowotny J., Cherif C. (2017). Approaches for Process and Structural Finite Element Simulations of Braided Ligament Replacements. J. Ind. Text..

[229] Higashiyama R., Sekiguchi H., Takata K., Endo T., Takamori Y., Takaso M. (2020). Arthroscopic Reconstruction of the Anterior Tibiotalar Ligament Using a Free Tendon Graft. Arthrosc. Tech..

[230] Lee K.I., Lee J.S., Kang K.T., Shim Y.B., Kim Y.S., Jang J.W., Moon S.H., D’Lima D.D. (2018). In Vitro and In Vivo Performance of Tissue-Engineered Tendons for Anterior Cruciate Ligament Reconstruction. Am. J. Sports Med..

[231] Pauly H.M., Kelly D.J., Popat K.C., Trujillo N.A., Dunne N.J., McCarthy H.O., Haut Donahue T.L. (2016). Mechanical Properties and Cellular Response of Novel Electrospun Nanofibers for Ligament Tissue Engineering: Effects of Orientation and Geometry. J. Mech. Behav. Biomed. Mater..

[232] Chen C.-H., Chen S.-H., Kuo C.-Y., Li M.-L., Chen J.-P. (2017). Response of Dermal Fibroblasts to Biochemical and Physical Cues in Aligned Polycaprolactone/Silk Fibroin Nanofiber Scaffolds for Application in Tendon Tissue Engineering. Nanomaterials.

[233] Sundararaj S., Slusarewicz P., Brown M., Hedman T. (2017). Genipin Crosslinker Releasing Sutures for Improving the Mechanical/Repair Strength of Damaged Connective Tissue. J. Biomed. Mater. Res. Part B Appl. Biomater..

[234] Atluri K., Chinnathambi S., Mendenhall A., Martin J.A., Sander E.A., Salem A.K. (2020). Targeting Cell Contractile Forces: A Novel Minimally Invasive Treatment Strategy for Fibrosis. Ann. Biomed. Eng..

[235] Gaut L., Duprez D., Gaut L., Duprez D., Reviews I. (2016). Tendon Development and Diseases. Wiley Interdiscip. Rev. Dev. Biol..

[236] Gouveia P.J., Hodgkinson T., Amado I., Sadowska J.M., Ryan A.J., Romanazzo S., Carroll S., Cryan S.A., Kelly D.J., O’Brien F.J. (2021). Development of Collagen-Poly (Caprolactone)-Based Core-Shell Scaffolds Supplemented with Proteoglycans and Glycosaminoglycans for Ligament Repair. Mater. Sci. Eng. C.

[237] Zhang B.Y., Xu P., Luo Q., Song G. (2021). Bin Proliferation and Tenogenic Differentiation of Bone Marrow Mesenchymal Stem Cells in a Porous Collagen Sponge Scaffold. World J. Stem Cells.

[238] Chen P., Li L., Dong L., Wang S., Huang Z., Qian Y., Wang C., Liu W., Yang L. (2021). Gradient Biomineralized Silk Fibroin Nanofibrous Scaffold with Osteochondral Inductivity for Integration of Tendon to Bone. ACS Biomater. Sci. Eng..

[239] Chen S., Wang J., Chen Y., Mo X., Fan C. (2021). Tenogenic Adipose-Derived Stem Cell Sheets with Nanoyarn Scaffolds for Tendon Regeneration. Mater. Sci. Eng. C.

[240] Mredha M.T.I., Guo Y.Z., Nonoyama T., Nakajima T., Kurokawa T., Gong J.P. (2018). A Facile Method to Fabricate Anisotropic Hydrogels with Perfectly Aligned Hierarchical Fibrous Structures. Adv. Mater..

[241] Laranjeira M., Domingues R.M.A., Costa-Almeida R., Reis R.L., Gomes M.E. (2017). 3D Mimicry of Native-Tissue-Fiber Architecture Guides Tendon-Derived Cells and Adipose Stem Cells into Artificial Tendon Constructs. Small.

[242] Zheng Z., Ran J., Chen W., Hu Y., Zhu T., Chen X., Yin Z., Heng B.C., Feng G., Le H. (2017). Alignment of Collagen Fiber in Knitted Silk Scaffold for Functional Massive Rotator Cuff Repair. Acta Biomater..

[243] Choi S., Choi Y., Kim J. (2019). Anisotropic Hybrid Hydrogels with Superior Mechanical Properties Reminiscent of Tendons or Ligaments. Adv. Funct. Mater..

[244] Bottagisio M., Lopa S., Granata V., Talò G., Bazzocchi C., Moretti M., Barbara Lovati A. (2017). Different Combinations of Growth Factors for the Tenogenic Differentiation of Bone Marrow Mesenchymal Stem Cells in Monolayer Culture and in Fibrin-Based Three-Dimensional Constructs. Differentiation.

[245] Younesi M., Akkus A., Akkus O. (2019). Microbially-Derived Nanofibrous Cellulose Polymer for Connective Tissue Regeneration. Mater. Sci. Eng. C.

[246] Domingues R.M.A., Chiera S., Gershovich P., Motta A., Reis R.L., Gomes M.E. (2016). Enhancing the Biomechanical Performance of Anisotropic Nanofibrous Scaffolds in Tendon Tissue Engineering: Reinforcement with Cellulose Nanocrystals. Adv. Healthc. Mater..

[247] Green E.C., Zhang Y., Li H., Minus M.L. (2017). Gel-Spinning of Mimetic Collagen and Collagen/Nano-Carbon Fibers: Understanding Multi-Scale Influences on Molecular Ordering and Fibril Alignment. J. Mech. Behav. Biomed. Mater..

[248] Sensini A., Cristofolini L., Focarete M.L., Belcari J., Zucchelli A., Kao A., Tozzi G. (2018). High-Resolution X-Ray Tomographic Morphological Characterisation of Electrospun Nanofibrous Bundles for Tendon and Ligament Regeneration and Replacement. J. Microsc..

[249] Sharifi-Aghdam M., Faridi-Majidi R., Derakhshan M.A., Chegeni A., Azami M. (2017). Preparation of Collagen/Polyurethane/Knitted Silk as a Composite Scaffold for Tendon Tissue Engineering. Proc. Inst. Mech. Eng. Part H J. Eng. Med..

[250] Mozafari M., Kargozar S., de Santiago G.T., Mohammadi M.R., Milan P.B., Koudehi M.F., Aghabarari B., Nourani M.R. (2018). Synthesis and Characterisation of Highly Interconnected Porous Poly(ε-Caprolactone)-Collagen Scaffolds: A Therapeutic Design to Facilitate Tendon Regeneration. Mater. Technol..

[251] Grier W.K., Iyoha E.M., Harley B.A.C. (2017). The influence of pore size and stiffness on tenocyte bioactivity and transcriptomic stability in collagen-GAG scaffolds. J. Mech. Behav. Biomed. Mater..

